# Evaluation of approaches for estimating the accuracy of genomic prediction in plant breeding

**DOI:** 10.1186/1471-2164-14-860

**Published:** 2013-12-06

**Authors:** Sidi Boubacar Ould Estaghvirou, Joseph O Ogutu, Torben Schulz-Streeck, Carsten Knaak, Milena Ouzunova, Andres Gordillo, Hans-Peter Piepho

**Affiliations:** 1 Bioinformatics Unit, Institute of Crop Science, University of Hohenheim, Fruwirthstrasse 23, 70599 Stuttgart, Germany; 2 KWS SAAT AG, 373555 Einbeck, Germany; 3 KWS-Lochow GMBH, Ferdinand-von-Lochow-Strasse 5, 29303 Bergen, Germany

**Keywords:** Genomic selection, Ridge-regression BLUP, Predictive accuracy, Predictive ability, Heritability, SNP markers, *Zea mays*, Cross-validation, Plant breeding

## Abstract

**Background:**

In genomic prediction, an important measure of accuracy is the correlation between the predicted and the true breeding values. Direct computation of this quantity for real datasets is not possible, because the true breeding value is unknown. Instead, the correlation between the predicted breeding values and the observed phenotypic values, called predictive ability, is often computed. In order to indirectly estimate predictive accuracy, this latter correlation is usually divided by an estimate of the square root of heritability. In this study we use simulation to evaluate estimates of predictive accuracy for seven methods, four (1 to 4) of which use an estimate of heritability to divide predictive ability computed by cross-validation. Between them the seven methods cover balanced and unbalanced datasets as well as correlated and uncorrelated genotypes. We propose one new indirect method (4) and two direct methods (5 and 6) for estimating predictive accuracy and compare their performances and those of four other existing approaches (three indirect (1 to 3) and one direct (7)) with simulated true predictive accuracy as the benchmark and with each other.

**Results:**

The size of the estimated genetic variance and hence heritability exerted the strongest influence on the variation in the estimated predictive accuracy. Increasing the number of genotypes considerably increases the time required to compute predictive accuracy by all the seven methods, most notably for the five methods that require cross-validation (Methods 1, 2, 3, 4 and 6). A new method that we propose (Method 5) and an existing method (Method 7) used in animal breeding programs were the fastest and gave the least biased, most precise and stable estimates of predictive accuracy. Of the methods that use cross-validation Methods 4 and 6 were often the best.

**Conclusions:**

The estimated genetic variance and the number of genotypes had the greatest influence on predictive accuracy. Methods 5 and 7 were the fastest and produced the least biased, the most precise, robust and stable estimates of predictive accuracy. These properties argue for routinely using Methods 5 and 7 to assess predictive accuracy in genomic selection studies.

## Background

Genomic selection (GS) is a method for predicting genomic breeding values using molecular markers covering the whole genome [1-3]. GS is fast becoming popular in plant and animal breeding [1,4,5], because of recent advances in high-throughput marker technologies and accompanying reduction in the costs of genotyping.

The performance of genomic selection (GS) procedures is often assessed by *k*-fold cross-validation (CV) [6]. Accurate evaluation of the performance of genomic selection is difficult in practice because true breeding values are typically unknown. As result, simulation modeling is often used to generate breeding values as a basis for assessing the accuracy of genomic prediction [1]. Once the true breeding values are available, the accuracy of genomic prediction can be expressed as the correlation between the true and the predicted breeding values. In this paper, we use simulated true breeding values to directly compute the true correlation (accuracy) between the true and the predicted breeding values $\left(r_{g,\hat{g}}\right)$ as a benchmark for evaluating the performance of seven contending methods. Four of the seven methods (Methods 1 to 4) first estimate heritability *H*^2^[7] and then divide the cross-validation sample correlation between the predicted breeding values ($\hat{g}$) and the observed phenotypic values (*p*), predictive ability, by the square root of heritability *H*^2^[8,9] to obtain an estimate of predictive accuracy $r_{\hat{g},p}/H$. The remaining three methods (Methods 5 to 7) estimate the predictive accuracy directly without having to first estimate heritability, even though Method 5 also estimates heritability. Here, we investigate the relative merits of the seven methods for estimating predictive accuracy using simulated breeding values. For five of the seven methods for estimating predictive accuracy (Methods 1, 2, 3, 4 and 6), we comparatively evaluate their predictive accuracies using three-fold cross-validation. Of the seven methods, two direct methods (Methods 5 and 6) and one indirect method (Method 4) for estimating predictive accuracy are proposed and described here for the first time whereas the remaining four methods were obtained from the literature. Methods 1 to 3 assume uncorrelated genotypes in the model for estimating heritability but assume correlated genotypes in the model for estimating predictive ability.

## Methods

We denote the standard deviation of a sample with *s* and that of a population with *σ* and the sample and population variance of the true genetic breeding values *g* with $s_{g}^{2}$ and $\sigma_{g}^{2}$, respectively. Further, we denote with *r*, $r_{\hat{g},p}$, *ρ* and *ρ*_*g*,*p*_ the sample correlation, the sample correlation between the BLUP of *g* and the observed “phenotypes” *p*, the population correlation and the population correlation between the true genetic breeding values *g* and the observed “phenotypes” *p*, respectively. Also, we use $r_{g,\hat{g}}$, $s_{g,\hat{g}}$, $s_{\hat{g}}^{2}$ and $s_{p}^{2}$ to denote the sample correlation between the true and the predicted genetic breeding values, the sample covariance between the true and the predicted breeding values, and the sample variance of the predicted breeding value and the phenotypic sample variance, respectively. In this paper, the sample will generally refer to a trial with *n* genotypes, real or simulated, assumed to have been obtained from an infinite population of genotypes.

### True correlation

The true correlation is given by the correlation between the true (*g*) and the predicted ($\hat{g}$) breeding values

(1) $$r_{g,\hat{g}}=\frac{s_{g,\hat{g}}}{\sqrt{s_{g}^{2}s_{\hat{g}}^{2}}},$$

where

(2) $$s_{g,\hat{g}}=\frac{1}{n-1}\sum_{i=1}^{n}\left(g_{i}-\bar{g}\right)\left({\hat{g}}_{i}-\bar{\hat{g}}\right)$$

is the covariance between the true and the predicted breeding values. Further,

(3) $$s_{g}^{2}=\frac{{\sum_{i=1}^{n}\left(g_{i}-\bar{g}\right)}^{2}}{n-1},$$

where $\bar{g}$ denotes the estimated mean of *g*_*i*_ (*i* = 1, …, *n*) and

(4) $$s_{\hat{g}}^{2}=\frac{{\sum_{i=1}^{n}\left({\hat{g}}_{i}-\bar{\hat{g}}\right)}^{2}}{n-1},$$

where $\bar{\hat{g}}$ denotes the estimated mean of ${\hat{g}}_{i}\left(i=1,\ldots ,n\right)$, are sample variances of the true and the predicted breeding values, respectively. We take the unobservable correlation $r_{g,\hat{g}}$ to be the main quantity of interest to the breeder or geneticist. Seven alternative procedures are evaluated, by simulation, regarding the accuracy and precision with which they are able to estimate $r_{g,\hat{g}}.$

### Two-stage approaches

We consider the case of a trial conducted in a single location. The analysis can be done in two stages [10]. The model for the observed plot data can be written as

(5) $$y=X_{1}\mu +f,$$

where *y* is the vector of the observed phenotypic values, μ is a vector containing the adjusted genotype means to be estimated from a model in which genotype enters as a fixed effect and *X*_1_ is an associated design matrix and *f* combines all the fixed, random design and error effects (replicates, blocks, etc.).

### The first stage of the two-stage approaches

At the first stage, means (*μ*) for the testcross genotypes are estimated using model (5) and submitted to the second stage. The adjusted means of the standard varieties are excluded from the dataset before submission to the second stage.

### The second stage of the two-stage approaches

The adjusted means from the first stage are used in the second stage to predict the true breeding values g. The second stage model is given by

(6) $${\hat{\mu }}_{i}=\varphi +g_{i}+e_{i},$$

where ${\hat{\mu }}_{i}$ is the adjusted mean of the *i*-th genotype (estimates of *μ*_*i*_ = *φ* + *g*_*i*_ from the first stage), *φ* is the general effect or mean, *g*_*i*_ is the random effect of the *i*-th genotype and *e*_*i*_ is the residual error associated with ${\hat{\mu }}_{i}$, *e* = (*e*_1_, …, *e*_*n*_)^*T*^ assumed to follow $e\sim N\left(0,R=I\sigma_{e}^{2}\right)$. The random vector *g* = (*g*_1_, …, *g*_*n*_)^*T*^ is modelled by a linear random regression on the SNP markers with random regression coefficients or marker effects *u* = (*u*_1_, …, *u*_*p*_)^*T*^ as

(7) g=Zu

where *Z* is the matrix of SNP marker covariates, $u\sim N\left(0,I_{p}\sigma_{u}^{2}\right)$, *I*_*p*_ is the *p* -dimensional identity matrix and $\sigma_{u}^{2}$ is the variance of marker effects.

Thus, we simulated the random SNP marker effects as random draws from a normal distribution with zero mean and variance $\sigma_{u}^{2}$. Genotyping of all the genotypes was done by SNP markers (275 for the AgReliant dataset and 11646 for the KWS Synbreed dataset, see below) and the information stored in a matrix *Z*_*snp*_ = {*z*_*ik*_}. The marker covariate *z*_*ik*_ for the *i*-th genotype (*i* = 1, 2, …, *n*) and the *k*-th marker (*k* = 1, 2, …, *p*) for biallelic SNP markers with alleles *A*_1_ and *A*_2_ was set to 1 for *A*_1_*A*_1_, -1 for *A*_2_*A*_2_ and to 0 for *A*_1_*A*_2_, *A*_2_*A*_1_ and missing values. Thus, the genotypic effect g has variance

(8) $$\text{var}\left(g=\mathrm{Zu}\right)=G=ZZ^{T}\sigma_{u}^{2},$$

where *Z*^*T*^ is the transpose of *Z*. Alternatively, for computing some of the heritability measures, we also fitted model (6) with $\text{var}\left(g\right)=G=I_{n}\sigma_{g}^{2}$, i.e., assuming that genotypic effects are uncorrelated for Methods 1 to 3, where $\sigma_{g}^{2}$ is the genetic variance and *I*_*n*_ is the *n*-dimensional identity matrix. In general, when fitting model 6, assuming a linear variance-covariance model for G as defined in (8) it can sometimes happen that the estimated marker variance (${\hat{\sigma }}_{u}^{2}$) is negative, yet it should not be. To ensure that the estimated ${\hat{\sigma }}_{u}^{2}$ is nonnegative it is necessary to specify a zero lower boundary constraint for ${\hat{\sigma }}_{u}^{2}$. This can be accomplished using the lower *b*=value-list option of the parms statement of the MIXED procedure when using the SAS system.

### Simulation of datasets

#### ***Assumed field design and model***

To simulate block and plot effects using variance components from the real maize (*Zea mays*) dataset provided by AgReliant we generated a dataset with 177 genotypes distributed over 10 incomplete blocks per replicate, each with 18 plots according to an alpha-design with two replicates. An appropriate model for this design must have an effect for the complete replicates, and another effect for the incomplete blocks, nested within replicates. We therefore simulated the field trial data according to an alpha design [11] using the model:

(9) $$y_{\text{ijk}}=\varphi +\gamma_{k}+b_{\mathrm{jk}}+g_{i}+e_{\text{ijk}}$$

where *y*_*ijk*_ is the yield of the *i*-th genotype in the *j*-th block nested within the *k*-th complete replicate, *φ* is the general effect or mean, *γ*_*k*_ is the fixed effect of the *k*-th complete replicate, *b*_*jk*_ is the random effect of the *j*-th block nested within the *k*-th complete replicate, *g*_*i*_ is the random effect of the *i*-th genotype, and *e*_*ijk*_ is the residual plot error associated with *y*_*ijk*_. We similarly simulated the block and plot effects using variance components estimated from the real maize dataset with 698 genotypes provided by KWS according to an alpha-design with two replicates based on model (9). The complete simulated datasets contained true genetic, block and plot effects. We used these datasets to compute the true correlation between the predicted and the true breeding values, true heritability as the square of the correlation between the predicted and the true breeding values and estimates of heritability for each of four different indirect methods. We considered four simulation scenarios defined by the parameters in Tables 1 and 2.

**Table 1:** The variance components for the AgReliant real maize data set estimated by RR-BLUP models assuming genotypes are correlated according to the linear variance model

**Variance components**	**Scenario 1**	**Scenario 2**
Marker ($\sigma_{u}^{2}$)	0.2019	0.2019/10
Block ($\sigma_{b}^{2}$)	69.9089	69.9089
Residual ($\sigma_{e}^{2}$)	48.6728	48.6728

**Table 2:** The variance components for the KWS-Synbreed real maize data set estimated by RR-BLUP models assuming genotypes are correlated according to the linear variance model

**Variance components**	**Scenario 3**	**Scenario 4**
Marker ($\sigma_{u}^{2}$)	0.005892	0.005892/10
Trial ($\sigma_{l}^{2}$)	11.8285	11.8285
Trial×Replicate ($\sigma_{r}^{2}$)	3.3231	3.3231
Trial×Replicate×Block ($\sigma_{b}^{2}$)	6.3148	6.3148
Tester×Non-genotyped $\left(\sigma_{g_{2}}^{2}\right)$ lines×GRP ($\sigma_{g}^{2}$)	34.5717	34.5717
Residual ($\sigma_{e}^{2}$)	53.8715	53.8715

#### ***Description of the real datasets and estimation of variance-components***

We used two real datasets to get marker information and estimates of the marker, block and error variance components, which we needed to simulate the true breeding values and phenotypic data, assuming correlated genotypes (Tables 1 and 2). For Scenarios 2 and 4 we divided the marker variance for Scenarios 1 and 3 by 10, respectively, to obtain smaller estimates of heritability (Tables 1 and 2).

### The AgReliant maize dataset

The first data set we used was a small dataset provided by AgReliant Genetics. It consisted of 177 doubled haploid maize lines derived from biparental crosses. The hybrid performance for kernel dry weight per plot of testcross genotypes was assessed with the same common tester using an unreplicated augmented trial design with incomplete blocks. Although the testcross genotypes were tested in six locations in one year, not all testcross genotypes were tested in each location. Furthermore, three to five incomplete blocks, each having one single row of plots, were used per location. Standard varieties connected the different blocks in the sense that they allowed estimation of the inter-block variance and separation of the block from the error variance. The standard varieties themselves were not used in predicting *g* but were used merely to facilitate analysis of the testcross genotypes. Markers with more than 20% missing values, or more than 5% heterozygous genotypes, or with minor allele frequency less than 2.5% were discarded [10]. We used the data for only one of the six locations with the RR-BLUP model to obtain variance components needed to simulate the random marker, block and plot effects for Scenarios 1 and 2. The selected location had a single unreplicated trial, 5 blocks, 2 checks and 177 lines, all of which were genotyped. Since the two checks had markers, just like all the other genotypes, they were treated in the exact same way as the other genotypes in the RR-BLUP model.

The RR-BLUP model assumed a linear variance model for the correlation between the genotypes:

(10) $$y_{\mathrm{ij}}=\varphi +b_{j}+g_{i}+e_{\mathrm{ij}},$$

where *y*_*ij*_ is the yield of the *i*-th genotype in the *j*-th block, *φ* is the general effect or mean, *b*_*j*_ is the random effect of the *j*-th block*,* the random vector *g* = (*g*_1_, …, *g*_*n*_)^*T*^ is modeled as in equation 7 and also has variance $\text{var}\left(g=\mathrm{Zu}\right)=ZZ^{T}\sigma_{u}^{2}$. The terms *Z*, *Z*^*T*^, *u* and $\sigma_{u}^{2}$ are defined as in equations 7 and 8 whereas *e*_*ij*_ is the residual plot error associated with *y*_*ij*_.

### The KWS-Synbreed maize dataset

The second data set was extracted for one location from a larger data set provided by KWS for the Synbreed project [12]. It had a total of 900 doubled haploid maize lines of which 698 testcrosses were genotyped while the remaining 202 were not, six hybrid checks and five line checks. The genotypes were crossed with four testers (Table 3). The testcross genotypes were tested using 9 trials each laid out according to a 10×10 lattice square design with incomplete blocks. Each trial had two replicates. There were a total of 1800 observations, 38 of which had no yield measurements.

**Table 3:** Definition of the variables in the KWS-Synbreed dataset used to compute covariance parameters used in the simulations for Scenarios 3 and 4

**Tester**	**GRP**	**Z1**	**Z2**	**GENA**	**GENB**	**Description of GENA**
T_0_	C_1_-C_6_	0	0	C_1_-C_6_	1	Hybrid checks 1-6
T_0_	nT	0	1	nT_1_- nT_4_	1	4 lines, not genotyped, unknown tester
T_0_	fT	0	1	fT_1_- fT_66_	1	16 lines, not genotyped, tested with a foreign tester
T_1_	G_0_	0	1	T1_1_-T1_66_	1	66 lines, not genotyped and tested to T1
T_2_	G_0_	0	1	T2_1_-T2_61_	1	61 lines, not genotyped and tested to T2
T_1_	G_1_	1	0	1-682	1-682	682 lines, genotyped and tested to T1 in group G1
T_1_	G_3_	1	0	683-698	683-698	16 lines, genotyped and tested to T1 in group G3

GRP = grouping factor for checks (C_1_-C_6_), testers (nT, fT) and groups (G_0_-G_3_) of genotyped lines. Z1=1 for genotyped lines and 0 otherwise. Z2=1 for non-genotyped lines and 0 otherwise. GENA denotes all the individual genotypes. GENB=GENA for genotyped lines and 1 otherwise. GENB helps specify the vector of random effects of genotyped lines with a length that matches the dimension of the covariance matrix of genotypes.

Fitting a linear variance model (7 and 8) to these data requires using a variance-covariance matrix of dimension *n*_1_ × *n*_1_, where *n*_1_ is the number of genotyped lines. The vector of effects of genotyped lines must therefore be of dimension *n*_1_. This presents a challenge for the KWS- data set because the vector of random effects of all the genotypes (*g*) contains both the vector of effects of the *n*_1_ genotyped lines (*g*_1_) plus the vector of the effects of the *n*_2_ non-genotyped lines (*g*_2_) and so has a larger dimension (*n*_1_+*n*_2_) than *n*_1_. To facilitate fitting the linear variance model for the genotyped lines we proceed as follows. First, we create a dummy variable in our dataset (*Z*_1*im*_, *i =*1*,…,n*_1_, *m*=1,…, 11 groups) equal to one for genotyped lines and zero otherwise. Second, we create a variable called GENA in the dataset with a unique level for each of the genotyped and the non-genotyped lines. Third, we create a second variable called GENB equal to GENA for the genotyped lines but equal to the level for any one of the genotyped lines for all the non-genotyped lines. Thus, the variable GENB has *n*_1_ levels, corresponding to the *n*_1_ genotyped lines. For example in Table 3, the variable GENB, whose effect is modelled by *g*_1_, has been set equal to 1, 2,…, 698 for the 698 genotyped lines and to 1, the label for the first genotyped line, for all the 202 ungenotyped lines. The genetic effect *g*_1*i*_ of the *i*-th genotyped line will be represented in the mixed model by the term *Z*_1*im*_*g*_1*i*_. This term will become zero for all the records corresponding to the non-genotyped lines, because for these records we have set *Z*_1*im*_=0. This ensures that the number of random genotypic effects to be predicted for *g*_1_ equals the dimension of the linear variance-covariance matrix (*n*_1_). The non-genotyped lines therefore make no contribution at all to the estimated variance-covariance matrix of the genotypes. They are, in other words, switched off.

The vector of random effects for the genotyped lines *g*_1_ is modelled by RR-BLUP as *g*_1_ = *Zu* with $\text{var}\left(g_{1}=\mathrm{Zu}\right)=ZZ^{T}\sigma_{u}^{2}$, where *Z* is the *n*_1_ × *p* design matrix for SNP markers for the *n*_1_ genotyped lines and *u* = (*u*_1_, …, *u*_*p*_)^*T*^ is the vector of *p* random SNP marker effects, with $u\sim N\left(0,I_{p}\sigma_{u}^{2}\right)$. *I*_*p*_ is the *p*-dimensional identity matrix and $\sigma_{u}^{2}$ is the variance of SNP marker effects.

We represent the genetic effects *g*_2*i*_ of the *i*-th non-genotyped line in a similar fashion as for *g*_1*i*_, i.e., we use the term *Z*_2*im*_*g*_2*i*_, where *Z*_2*i*_ is a dummy variable that is equal to one for all the non-genotyped lines and equal to zero for all the genotyped lines. The effects *g*_2*i*_ are assumed to be independent normally and distributed with variance $\sigma_{g_{2}}^{2}$.

The following mixed model, assuming that the genotyped lines are correlated according to the RR-BLUP model (10), was used to estimate the variance components used in the simulations for Scenarios 3 and 4:

(11) $$\begin{array}{ll} y_{\text{ijklmn}}= & \varphi +t_{l}+r_{\mathrm{kl}}+b_{\text{jkl}}+\delta_{m}+\tau_{n}\\ & +Z_{1\mathrm{im}}{g_{1}}_{\mathrm{im}}+Z_{2\mathrm{im}}{g_{2}}_{\mathrm{im}}+e_{\text{ijklmn}}, \end{array}$$

where *y*_*ijklmn*_ is the response of the *i*-th genotype in the *j*-th block nested within the *k*-th replicate in the *l*-th trial in the *m*-th group tested against the *n*-th tester. *φ* is the general effect, *t*_*l*_ is the random effect of the *l*-th trial, assumed iid $N\left(0,\sigma_{t}^{2}\right)$, *r*_*kl*_ is the random effect of the *k*-th replicate nested within the *l*-th trial, assumed iid $N\left(0,\sigma_{r}^{2}\right)$, *b*_*jkl*_ is the random effect of the *j*-th block nested within the *k*-th replicate in the *l*-th trial, assumed iid $N\left(0,\sigma_{b}^{2}\right)$, *δ*_*m*_ is the fixed effect of the *m*-th group of checks, testers and genotypes, *τ*_*n*_ denotes the effect of the *n*-th tester (Table 3) and *e*_*ijklmn*_ is the residual plot error associated with *y*_*ijklmn*_ and is assumed to be iid $N\left(0,\sigma_{e}^{2}\right)$, where $\sigma_{e}^{2}$ is the error variance.

To implement the model using the REML package PROC MIXED of the SAS System [13], the random genotypic effects were coded using the variables defined in Table 3. The random genotypic effect of the *i-*th genotyped line in the *m*-th group, *Z*_1*im*_*g*_1*im*_, was coded as (Z1*TS*GRP*GENB) using the variables tester (TS), group (GRP), genotypes (GENB), and Z1, where the last variable was specified as a quantitative variable, while the first three variables were declared as categorical variables (using the CLASS statement). The variable Z1 corresponds to the switch variable *Z*_1*im*_ in the model (11). The random effect *Z*_2*im*_*g*_2*im*_ of the *i*-th non-genotyped line in the *m*-th group was coded as (Z2*TS*GRP*GENA) using the variables tester (TS), group (GRP) and genotypes (GENA) described in Table 3. Z2 is a second quantitative variable corresponding to the switch variable *Z*_2*im*_.

### Estimating predictive accuracy from predictive ability and heritability

Four of the seven approaches indirectly estimate predictive accuracy $\left(r_{\hat{g},p}/H\right)$ as the correlation between the predicted genetic and phenotypic values $\left(r_{\hat{g},p}\right)$, called predictive ability, divided by the square root of heritability (*H*^2^) [14], separately for each of 15 three-fold cross-validation replicates. Predictive ability can be estimated from cross-validation as

(12) $$r_{\hat{g},p}=\frac{s_{\hat{g},p}}{\sqrt{s_{\hat{g}}^{2}s_{p}^{2}}}.$$

A key assumption of the approach is that $s_{\hat{g},p}=s_{g,\hat{g}}$[8], which implies that the genotypic effect estimate $\hat{g}$ is not correlated with environmental components in the phenotypic value *p*. This suggests [8] that in practice we can replace (1) with

(13) $$r_{g,\hat{g}}=\frac{s_{\hat{g},p}}{\sqrt{s_{\hat{g}}^{2}s_{g}^{2}}}.$$

The other assumption is that $s_{g}^{2}=H^{2}s_{p}^{2}$, so that

(14) $$r_{g,\hat{g}}=\frac{s_{\hat{g},p}}{\sqrt{s_{\hat{g}}^{2}s_{g}^{2}}}=\frac{s_{\hat{g},p}}{H\sqrt{s_{\hat{g}}^{2}s_{p}^{2}}}=\frac{r_{\hat{g},p}}{H}.$$

An important question is how to estimate heritability *H*^2^. The fact that the definition of $\hat{g}$ used in $r_{g,\hat{g}}$ requires a marker-based model for *g* suggests that the same model should be used for defining heritability *H*^2^. The problem in practice is that the true model is not known, so that different methods for genomic selection (GS) are usually applied and their predictions compared empirically via CV [15]. To make any progress, some model must be chosen for defining predictive accuracy, and if the chosen model is close to the model for some GS method, then that same method would potentially be preferred for the estimation of predictive accuracy. Moreover, some methods for GS do not have an explicit underlying model. This is the case for some methods, for example, in the machine learning realm.

The difficulty in choosing a model for heritability *H*^2^ makes it hard to devise an unambiguous definition for *H*^2^. Thus, any estimate of predictive accuracy should be taken only as a rough indication of precision. We suggest that the model underlying ridge regression BLUP be used to define heritability *H*^2^. This is because this method is the most commonly used one for GS and has been shown to have good properties. It is based on a specific mixed model, so an estimate of *H*^2^ can be obtained in various ways [16].

### Cross-validation

We used cross-validation (CV) to obtain an estimate of the correlation between the predicted breeding values and the observed phenotypic values $\left(r_{\hat{g},p}\right)$, which we needed to compute predictive accuracy $\left(r_{g,\hat{g}}\right)$ for Methods 1, 2, 3, 4 and 6. We used a three-fold cross-validation to evaluate predictive accuracy for both datasets because of the small number of genotypes (177) in the AgReliant dataset. The dataset with the adjusted means for the testcross genotypes was split into three random subsamples, one of which was held out as a validation set at a time. The remaining two subsamples were combined into one training set. The three-fold CV procedure was replicated five times, yielding a total of 15 replicate datasets. We then fitted a ridge regression model (7) to each of the 15 replicate validation and training sets. We next computed predictive ability, the correlation between the predicted breeding values and the phenotypic values $r_{\hat{g},p}$ across all the genotypes. This procedure was repeated for each of the 1000 datasets simulated for each of the four scenarios. The correlation between the predicted and the true breeding values $r_{g,\hat{g}}$, the predictive accuracy, was computed by dividing predictive ability $r_{\hat{g},p}$ by the square root of estimated heritability for each of the four indirect methods. The estimates of predictive accuracy were compared using the simulated true breeding values. Moreover, we directly computed predictive accuracy using Methods 5, 6 and 7 as described below. Table 4 summarizes the key properties of the seven methods.

**Table 4:** Summary of the main properties of the seven methods

**Method**	**Estimates heritability?**	**Requires heritability to estimate predictive accuracy?**	**Model for heritability assumes uncorrelated genotypes?**	**Model for predictive ability assumes correlated genotypes?**	**Requires cross validation?**
1	Yes	Yes	Yes	Yes	Yes
2	Yes	Yes	Yes	Yes	Yes
3	Yes	Yes	Yes	Yes	Yes
4	Yes	Yes	Yes	Yes	Yes
5	Yes	No	-	-	No
6	No	No	-	Yes	Yes
7	No	No	-	-	No

Methods 1 to 4 that require heritability to estimate predictive accuracy are called indirect methods whereas Methods 5 to 7 that do not are called direct methods in the text. The symbol (-) means the question is not applicable for a particular model.

### Methods for estimating predictive accuracy

We used the following five methods (Methods 1 to 5) to estimate heritability (Table 5). The first approach is the standard method for estimating heritability that is commonly used by plant breeders [12]. The second and the third approaches are modifications of the *ad hoc* measure based on BLUE and BLUP [16]. The fourth approach is based on a new proposal for estimating heritability. This approach uses the ratio of the expected value of the genetic variance to the expected value of phenotypic variance. The fifth approach is our second new method for estimating heritability without cross-validation using similar ideas to those used in computing the *ad hoc* measures of *H*^2^ (Table 5). Methods 1 to 3 assume that the genotypes are not correlated, while Methods 4 and 5 assume correlated effects according to the model underlying the RR-BLUP. We then used the quantity $r_{\hat{g},p}/H$ to compute predictive accuracy, where *H* is the square root of heritability computed using each of the first four methods (Methods 1 to 4) only. This is because even though Method 5 also computes heritability, it calculates predictive accuracy directly, similar to Methods 6 and 7. The three Methods 5, 6 and 7 were thus used to estimate predictive accuracy directly without first dividing predictive ability by the square root of heritability. The three direct methods assume correlated effects of genotypes according to the model underlying RR-BLUP.

**Table 5:** Descriptive statistics for the estimated true heritability for all the datasets out of a possible total of 1000 for which an estimate of heritability was available for all the five methods in each scenario

			**Methods**				
		**M0**	*M1(15)	**M2(16)**	**M3(17)**	**M4(21)**	**M5(24)**
**Scenario**	^**†**^**Statistic**	$H_{\text{true}}^{2}$	${\hat{H}}_{m1}^{2}$	${\hat{H}}_{m2}^{2}$	${\hat{H}}_{m3}^{2}$	${\hat{H}}_{m4}^{2}$	${\hat{H}}_{m5}^{2}$
1	${\hat{H}}^{2}=0$	0	0	0	0	0	0
	MIN	0.56	0.09	0.16	0.16	0.16	0.50
	MAX	0.82	0.50	0.66	0.66	0.55	0.80
	MEAN	0.71	0.32	0.48	0.48	0.34	0.67
	STD	0.04	0.06	0.08	0.08	0.06	0.05
	MSD	0.000	0.160	0.061	0.061	0.143	0.004
2	${\hat{H}}^{2}=0$	0	152	152	0	0	0
	MIN	0.09	0.00	0.00	0.00	0.00	0.00
	MAX	0.73	0.30	0.46	0.46	0.28	0.63
	MEAN	0.42	0.09	0.15	0.18	0.08	0.33
	STD	0.11	0.07	0.11	0.10	0.05	0.11
	^†^MSD	0.000	0.128	0.094	0.083	0.128	0.025
3	${\hat{H}}^{2}=0$	0	0	0	0	0	0
	MIN	0.66	0.35	0.33	0.34	0.28	0.64
	MAX	0.79	0.62	0.61	0.61	0.53	0.78
	MEAN	0.73	0.51	0.49	0.50	0.40	0.72
	STD	0.02	0.04	0.04	0.04	0.03	0.02
	MSD	0.000	0.051	0.057	0.055	0.110	0.001
4	${\hat{H}}^{2}=0$	0	0	0	0	0	0
	MIN	0.36	0.00	0.00	-0.55	0.02	0.23
	MAX	0.63	0.32	0.31	0.31	0.15	0.52
	MEAN	0.52	0.14	0.13	0.13	0.07	0.39
	STD	0.04	0.07	0.06	0.07	0.02	0.05
	MSD	0.000	0.151	0.155	0.152	0.202	0.020

Methods 1 to 4 but not 5 use cross-validation. M0 is the square of the true correlation between the predicted and the true simulated breeding values used as the benchmark for assessing the estimated heritability.

^†^MSD=Mean squared deviation and *H*^2^ = 0 is the number of datasets for which the estimated heritability was zero. *The number of the equation used in the text is in parenthesis.

**Method 1:** Standard measure

Plant breeders often compute heritability for a single trial using

(15) $$H_{m1}^{2}=\frac{\sigma_{g}^{2}}{\sigma_{g}^{2}+\sigma_{e}^{2}/r},$$

where $\sigma_{g}^{2}$ is the genetic variance, *r* is the number of replicates and *σ*^2^_*e*_ is the variance of plot error [12]. This estimator is valid for randomized complete block designs, but is an *ad hoc* approximation for incomplete block designs. Also, the estimator is not applicable with spatial methods of analysis [17].

**Method 2:** A measure proposed by [16] that uses the BLUEs and is computed as

(16) $$H_{m2}^{2}=\frac{{\sigma_{g}}^{2}}{{\sigma_{g}}^{2}+\bar{\upsilon }/2},$$

where $\bar{\upsilon }$ is the mean variance of a difference of two adjusted genotypic means (BLUE) and $\sigma_{g}^{2}$ is the genetic variance estimated from (6) assuming independent genotypic effects.

**Method 3:** An *ad hoc* measure proposed by [18] that is based on BLUP assuming independent genotypic effects and is computed as

(17) $$H_{m3}^{2}=1-\frac{{\bar{\upsilon }}_{\text{BLUP}}}{2{\sigma_{g}}^{2}},$$

where ${\bar{\upsilon }}_{{}_{\text{BLUP}}}$ is the mean variance of a difference of the BLUP of two genotypic effects ${\hat{g}}_{i}$. We used the BLUP of *μ*_*i*_ = *φ* + *g*_*i*_ as the phenotypic data in the mixed model for Method 3. Further details on the computation of ${\bar{\upsilon }}_{{}_{\text{BLUP}}}$ and $\bar{\upsilon }$ are in Additional file 1.

**Method 4:** A new proposed measure for estimating heritability

The sample variance $s_{g}^{2}$ of the true genetic breeding value *g*_1_ (3) can be written as

(18) $$s_{g}^{2}=\frac{1}{n-1}\sum_{i=1}^{n}{\left(g_{i}-\bar{g}\right)}^{2}=g^{T}P_{u}g,$$

where $P_{u}=\frac{1}{n-1}\left(I_{n}-\frac{1}{n}J_{n}\right)$, *I*_*n*_ is the *n*-dimensional identity matrix and *J*_*n*_ = *n* × *n* is a square matrix of ones. In a similar way, we may represent the phenotypic sample variance as

(19) $$s_{p}^{2}=\frac{1}{n-1}\sum_{i=1}^{n}{\left(p_{i}-\bar{p}\right)}^{2}=p^{T}P_{u}p,$$

where $p=\hat{\mu }={\left({\hat{\mu }}_{1},{\hat{\mu }}_{2},\ldots ,{\hat{\mu }}_{n}\right)}^{T}$ is the vector of observed phenotypes. The observed phenotype ${\hat{\mu }}_{i}$ of the *i*-th genotype is the adjusted mean computed for this genotype for a single location.

We cannot compute the sample variance of *g* for real data because these are not observed. But if we assume a mixed model with linear variance structure for *g* taking the form $\text{var}\left(g\right)=G=ZZ^{T}\sigma_{u}^{2}$, where *g* is the vector of *g*_*i*_ of all tested entries (*i* = 1, …, *n*), and $\sigma_{u}^{2}$ is the SNP marker variance, then we can compute the expected value of the sample variance of *g*_*i*_ in equation 18. From standard results on the expected value of quadratic forms [12] we have

(20) $$E\left(s_{g}^{2}\right)=\text{trace}\left(P_{u}G\right).$$

Thus, we may define heritability as the expected genetic sample variance $s_{g}^{2}$ over the expected phenotypic sample variance:

(21) $$H_{m4}^{2}=\frac{E\left(s_{g}^{2}\right)}{E\left(s_{p}^{2}\right)}.$$

The expected value $E\left(s_{p}^{2}\right)$ is now derived. The variance of phenotypes (i.e., adjusted means *p*) is given by

$$\text{var}\left(p\right)=V=G+R,$$

where *R* is the variance-covariance matrix of the error term in equation (6). Therefore

(22) $$E\left(s_{p}^{2}\right)=\text{trace}\left(VP_{u}\right)=E\left(s_{g}^{2}\right)+\text{trace}\left(RP_{u}\right).$$

An estimate of this is easy to compute, as is an estimate of $\left(s_{g}^{2}\right)$, by plugging in estimates for the variance-components in *G* and *R*. For illustration of the new Method 4, we consider three special cases in Additional file 2.

**Method 5:** A new direct method for estimating $r_{g,\hat{g}}$

This method uses the RR-BLUP of *g* as the “phenotype” to compute an alternative estimator of predictive accuracy unlike that produced by the methods that require cross-validation and use the adjusted means as the phenotypes. Heritability can be computed as the square root of the estimated predictive accuracy.

Using equation 7, we have

$$\text{BLUP}\left(g=\mathrm{Zu}\right)=\hat{g}=GV^{-1}\left(p-1\hat{\varphi }\right)$$

and $\hat{\varphi }={\left(1^{T}V^{-1}1\right)}^{-1}1^{T}V^{-1}p.$ Thus, *BLUP*(*g* = *Zu*) = *GV*^- 1^*Qp*, where *Q* = *I* - 1(1^*T*^*V*^- 1^1)^- 1^1^*T*^*V*^- 1^. Or in an even more compact form $\text{BLUP}\left(g=\mathrm{Zu}\right)=\hat{g}=\mathrm{Cp}$ with *C* = *GV*^- 1^*Q*.

Now consider the sample correlation of the true genotypic value *g* = *Zu* and its estimator $\hat{g}$, the BLUP of *g*. The sample covariance $s_{g,\hat{g}}$ is given by

$$s_{g,\hat{g}}=g^{T}P_{u}\hat{g}=g^{T}P_{u}\mathrm{Cp},$$

where *P*_*u*_ is defined as in equation 18. We cannot compute this sample covariance directly, because *g* is not observable. But we can compute its expected value as follows:

$$\begin{array}{ll} E\left(s_{g,\hat{g}}\right) & =E\left(g^{T}P_{u}\mathrm{Cp}\right)=E\left(g^{T}P_{u}\mathrm{Cg}\right)\\ & =E\left[\text{trace}\left(g^{T}P_{u}\mathrm{Cg}\right)\right]=E\left[\text{trace}\left(P_{u}\mathrm{Cg}g^{T}\right)\right]\\ & =\text{trace}\left[E\left(P_{u}\mathrm{Cg}g^{T}\right)\right]=\text{trace}\left(P_{u}\mathrm{CG}\right) \end{array}$$

In the same vein, we find for the sample variances of the true (*g*) and the predicted ($\hat{g}$) breeding values:

$$s_{g}^{2}=g^{T}P_{u}g$$

$$E\left(s_{g}^{2}\right)=\text{trace}\left(P_{u}G\right)$$

$$s_{\hat{g}}^{2}=p^{T}C^{T}P_{u}P_{u}\mathrm{Cp}=p^{T}C^{T}P_{u}\mathrm{Cp}$$

$$E\left(s_{\hat{g}}^{2}\right)=\text{trace}\left(C^{T}P_{u}\mathrm{CV}\right)$$

The sample true correlation from equation 1 is then given by

$$r_{g,\hat{g}}=\frac{s_{g,\hat{g}}}{\sqrt{s_{g}^{2}s_{\hat{g}}^{2}}}.$$

We want to estimate the expected value of this correlation. Approximately, we have

(23) $$E\left(r_{g,\hat{g}}\right)\approx \frac{E\left(s_{g,\hat{g}}\right)}{\sqrt{E\left(s_{g}^{2}\right)E\left(s_{\hat{g}}^{2}\right)}}.$$

Note that the correlation involves a function of three correlated random variables ($s_{\hat{g},g},s_{g}^{2},s_{\hat{g}}^{2}$) and we must acknowledge that the expected value of a function of random variables is not usually exactly equal to the same function evaluated at the expected values of the random variables [19]. Some improvement of the approximation may be possible using the delta method, but we will not pursue this here. Instead, the closeness of the approximation (23) will be investigated in our simulations. From a practical point of view, the advantage of (23) is its simplicity. With this approximation, we may replace the expected values with their explicit expressions to yield the estimated predictive accuracy:

(24) $$E\left(r_{g,\hat{g}}\right)\approx H_{m5}=\frac{\text{trace}\left(P_{u}\mathrm{CG}\right)}{\sqrt{\text{trace}\left(P_{u}G\right)\text{trace}\left(C^{T}P_{u}\mathrm{CV}\right)}}.$$

**Method 6:** A further new direct method for estimating

$$r_{g,\hat{g}}$$

Our objective is to estimate $r_{g,\hat{g}}$ by evaluating (14). It is straightforward to compute $s_{\hat{g},p}$ and $s_{\hat{g}}^{2}$ in (14) directly from the data (*p*) and the predicted breeding values ($\hat{g}$). The only difficulty is the estimation of $s_{g}^{2}$. If we could observe the *g* of all entries, we would compute their sample variance $s_{g}^{2}$, just as we compute the sample variance $s_{\hat{g}}^{2}$ and the sample covariance $s_{\hat{g},p}$. Note that we similarly computed the sample correlation between the predicted breeding values $\hat{g}$ and the observed phenotypic values *p* for each cross-validation replicates. Our proposed estimator becomes

(25) $${\hat{r}}_{g,\hat{g},m6}=\frac{s_{\hat{g},p}}{\sqrt{s_{\hat{g}}^{2}\times E\left(s_{g}^{2}\right)}},$$

where $E\left(s_{g}^{2}\right)$ was computed using equation 23. A Problem with this method as well as with Methods 1, 2, 3 and 4 is that ${\hat{r}}_{g,\hat{g},\mathrm{m6}}$ can exceed one. We have therefore presented values of ${\hat{r}}_{g,\hat{g},m6}$ truncated at 1 in the main body of the paper and the untruncated values in Additional file 3 (Table 6 and Additional file 3: Table S1).

**Table 6:** Descriptive statistics for predictive accuracy (estimates less than 0 were set to 0 whereas estimates greater than 1 were set to 1) by scenario

		**Methods**							
**Scenario**	^**†**^**Statistic**	**M0**	^*^M1(15)	**M2(16)**	**M3(17)**	**M4(21)**	**M5(24)**	**M6(25)**	**M7(35)**
		$r_{g,\hat{g}}$	$\frac{r_{\hat{g},p}}{{\hat{H}}_{m1}}$	$\frac{r_{\hat{g},p}}{{\hat{H}}_{m2}}$	$\frac{r_{\hat{g},p}}{{\hat{H}}_{m3}}$	$\frac{r_{\hat{g},p}}{{\hat{H}}_{m4}}$	${\hat{H}}_{m5}$	$r_{g,\hat{g},m6}$	${\hat{\rho }}_{m7}$
1	N	1000	1000	1000	1000	1000	1000	1000	1000
	MIN	0.750	0.327	0.265	0.265	0.384	0.707	0.316	0.750
	MEAN	0.843^c^	0.877^a^	0.727 ^e^	0.727^e^	0.858^b^	0.819^d^	0.663^f^	0.840^c^
	MAX	0.908	1.000	1.000	1.000	1.000	0.893	0.884	0.899
	STD	0.024	0.109	0.104	0.104	0.093	0.028	0.084	0.023
	MSD	0.000	0.013	0.025	0.025	0.009	0.002	0.040	0.001
	Q1	0.829	0.807	0.661	0.661	0.802	0.803	0.612	0.826
	Median	0.846	0.890	0.723	0.724	0.862	0.822	0.665	0.841
	Q3	0.860	0.983	0.793	0.793	0.923	0.839	0.716	0.856
2	N	839	839	839	839	839	839	839	839
	MIN	0.31	0.00	0.00	0.00	0.00	0.06	0.00	0.08
	MEAN	0.65^a^	0.63^c^	0.50^e^	0.50^e^	0.64^ab^	0.58^d^	0.46^f^	0.64^bc^
	MAX	0.85	1.00	1.00	1.00	1.00	0.79	1.00	0.82
	STD	0.09	0.29	0.26	0.26	0.26	0.10	0.20	0.09
	MSD	0.000	0.083	0.092	0.092	0.069	0.02	0.081	0.011
	Q1	0.61	0.42	0.31	0.31	0.47	0.53	0.33	0.59
	Median	0.66	0.64	0.48	0.48	0.68	0.59	0.47	0.65
	Q3	0.71	0.91	0.67	0.67	0.85	0.64	0.59	0.70
3	N	1000	1000	1000	1000	1000	1000	1000	1000
	MIN	0.81	0.60	0.61	0.61	0.69	0.80	0.54	0.78
	MEAN	0.85^a^	0.72^c^	0.73^c^	0.73^c^	0.81^b^	0.85^a^	0.64^d^	0.81^b^
	MAX	0.89	0.88	0.90	0.89	0.96	0.88	0.78	0.84
	STD	0.01	0.04	0.04	0.04	0.04	0.01	0.04	0.01
	MSD	0.0000	0.0193	0.0169	0.0176	0.0036	0.0002	0.0477	0.0017
	Q1	0.85	0.69	0.70	0.70	0.79	0.84	0.61	0.81
	Median	0.85	0.72	0.73	0.73	0.81	0.85	0.64	0.81
	Q3	0.86	0.75	0.76	0.76	0.84	0.85	0.66	0.82
4	N	955	955	955	955	955	955	955	955
	MIN	0.60	0.14	0.14	0.14	0.15	0.48	0.24	0.52
	MEAN	0.72^a^	0.32^e^	0.33^e^	0.33^e^	0.36^d^	0.62^c^	0.63^b^	0.64^b^
	MAX	0.79	0.52	0.53	0.52	0.56	0.72	0.93	0.72
	STD	0.03	0.06	0.06	0.06	0.07	0.04	0.09	0.03
	MSD	0.000	0.160	0.157	0.158	0.130	0.012	0.015	0.007
	Q1	0.70	0.29	0.29	0.29	0.32	0.60	0.57	0.62
	Median	0.72	0.32	0.33	0.33	0.37	0.62	0.64	0.64
	Q3	0.74	0.36	0.37	0.37	0.41	0.65	0.70	0.66

All the methods except 5 and 7 use cross-validation. M0 is the correlation between the predicted and the true simulated breeding values used as the benchmark for assessing the estimated predictive accuracy. N is the number of data sets out of a possible total of 1000 for which estimates were available for all the seven methods. Means for pairs of methods within each scenario with the same superscript letter are not significantly different at the 5% level of significance based on the *t*-test.

^†^MSD=Mean squared deviation, Q1 is the lower quartile and Q3 is the upper quartile. * The number of the equation used in the text is in parenthesis.

**Method 7:** This method is commonly used in animal breeding [20-22]

We used the linear mixed model:

(26) y=χβ+ζu+e,

where *X* is the design matrix for the fixed effects, *β* is the vector of regression coefficients for the fixed effects, $u\sim N\left(0,\tilde{G}=\iota_{p}\sigma_{u}^{2}\right)$ is the random marker effects with variance $\sigma_{u}^{2}$, the residual errors *e* = (*e*_1_, …, *e*_*n*_)^*T*^ are assumed to follow $e\sim N\left(0,R=I_{n}\sigma_{e}^{2}\right)$ with variance $\sigma_{e}^{2}$ and Z is the marker matrix. The random vector *g* = (*g*_1_, …, *g*_*n*_)^*T*^ is obtained from a linear regression on the random marker (SNP) effects *u*_*k*_, i.e. *u* = (*u*_1_, …, *u*_*p*_)^*T*^ as

(27) g=Zu.

A common approach to the evaluation of predictive accuracy (*ρ*) in animal breeding is the use of the squared correlation between the true and the predicted breeding values (*ρ*^2^), called reliability [20]. We used the approach of [20] as implemented by [16]. The calculation of *ρ*^2^ requires a solution to the mixed model equations [21] given by [16]

(28) $$\begin{array}{ll} \left(\begin{array}{c} \hat{\beta }\\ \hat{u} \end{array}\right) & ={\left(\begin{array}{cc} \chi^{T}R^{-1}\chi & \chi^{T}R^{-1}\zeta \\ \zeta^{T}R^{-1}\chi & \zeta^{T}R^{-1}\zeta +G^{-1} \end{array}\right)}^{-}\left(\begin{array}{c} \chi^{T}R^{-1}y\\ \zeta^{T}R^{-1}y \end{array}\right)\\ & =\left(\begin{array}{cc} C_{11} & C_{12}\\ C_{21} & C_{22}\; \end{array}\right)\;\left(\begin{array}{c} \;\chi^{T}R^{-1}y\\ \zeta^{T}R^{-1}y \end{array}\right), \end{array}$$

where ()^-^ denotes a generalized inverse of the coefficient matrix of the MME [23]. If the variance-covariance matrix of the random effects *u* and the genetic effects *g* = *Zu* is given by

(29) $$\text{var}\left(\begin{array}{c} g\\ u \end{array}\right)=\left(\begin{array}{cc} D & F\\ F^{T} & G \end{array}\right),$$

where $D=ZZ^{T}\sigma_{u}^{2}$ and $F=Z\sigma_{u}^{2},$ then it follows that [24]

(30) $$\text{BLUP}\left(g\right)=FG^{-1}\hat{u}=Z\hat{u},$$

where

$$\hat{u}=\text{BLUP}\left(u\right).$$

The distribution of *g* and $\hat{g}$ is then multivariate normal with zero mean and variance-covariance matrix

(31) $$\text{var}\left(\begin{array}{c} g\\ \hat{g} \end{array}\right)=\left(\begin{array}{cc} ZZ^{T}\sigma_{u}^{2} & \mathrm{ZM}G^{-1}Z^{T}\sigma_{u}^{2}\\ \mathrm{ZM}G^{-1}Z^{T}\sigma_{u}^{2} & \mathrm{ZM}G^{-1}Z^{T}\sigma_{u}^{2} \end{array}\right),$$

where $M=\text{var}\left(\hat{u}\right)$[25]. The REML estimate of *M* can be computed from a mixed model package by noting that

(32) $$\text{var}\left(\hat{u}\right)=M=G-C_{22}.$$

After substituting for *M*, equation 32 reduces to

(33) $$\text{var}\left(\begin{array}{c} g\\ \hat{g} \end{array}\right)=\left(\begin{array}{cc} \zeta \zeta^{T}\sigma_{u}^{2} & \zeta \zeta^{T}\sigma_{u}^{2}-ZC_{22}Z^{T}\\ \zeta \zeta^{T}\sigma_{u}^{2}-ZC_{22}Z^{T} & \zeta \zeta^{T}\sigma_{u}^{2}-ZC_{22}Z^{T} \end{array}\right),$$

from which the reliability $\rho_{i}^{2}$ for each genotype is calculated as

(34) $${\hat{\rho }}_{i}^{2}=\frac{{\left(\text{cov}\left(g_{i},{\hat{g}}_{i}\right)\right)}^{2}}{\text{var}\left(g_{i}\right).\text{var}\left({\hat{g}}_{i}\right)},$$

where we extract only the corresponding diagonal elements from the block matrices var(*g*), $\text{var}\left(\hat{g}\right)$ and $\text{cov}\left(g,\hat{g}\right)$. The reliability of all genotypes in each dataset is then estimated by

(35) $${\hat{\rho }}_{{}^{m7}}^{2}=\frac{1}{n}\sum_{i=1}^{n}{\hat{\rho }}_{i}^{2}$$

and the accuracy by its square root, where *n* is the total number of genotypes in the data set. The SAS (version 9.3) code used to simulate the phenotypic data and fit all the seven models is provided in Additional file 4.

### Evaluation of simulated data

We used the two-stage analysis and the methods described above to estimate the correlations between the predicted and the true breeding values $r_{g,\hat{g}}$. We computed the true heritability as the square of the correlation between the predicted and the true breeding values and estimated heritability using the five different approaches. We then computed the ratio of the expected value of the genetic variance to the expected value of the phenotypic variance and used this to compute heritability based on the new proposed method (Method 4) for estimating heritability. Moreover, we estimated the adjusted least square means of genotypes from the first stage using simulated data. The adjusted least square means were used in the second stage in cross-validations as the phenotypic data. Also, the variance-covariance matrix of the adjusted means was used to compute an *ad hoc* measure of heritability according to equation 16. Because the KWS maize dataset had many genotypes (n = 698) the mixed models were computationally very demanding to fit. So, for example, the slowest method, Method 6, took only 17.5 hrs to fit all the 1000 small data sets in one scenario in SAS Version 9.3 running on a 64-bit Windows 7 workstation with 8 GB RAM and an Intel Core Quad 2.66, but it took 192 hrs to fit the same model to 1000 large data sets. Hence, we first estimated the start values for the variance-components of the mixed models using the hpmixed procedure of SAS to enhance computational efficiency.

### Comparing heritabilities

We computed true heritability as the square of the correlation between the predicted and the simulated true breeding values:

(36) $$H_{\text{true}}^{2}={\left(r_{g,\hat{g}}\right)}^{2}$$

To compare the true heritabilities *H*^2^ with their estimates computed using each of the four different indirect methods (Methods 1, 2, 3, and 4) and Method 5 we computed the mean squared deviation (MSD) of each estimate from the true heritability for each simulated dataset and method combination as

(37) $$\text{MSD}=\sum_{j=1}^{N}\frac{{\left({\hat{H}}_{j}^{2}-{\left(r_{g,\hat{g},j}\right)}^{2}\right)}^{2}}{N}$$

where *N* is the total number of the simulated datasets and *j=* (1, 2, …, *N*) denotes the *j-*th simulated data set*.* Moreover, we computed descriptive statistics for the true and estimated heritabilities.

### Comparing predictive accuracies

**Figure 1:**
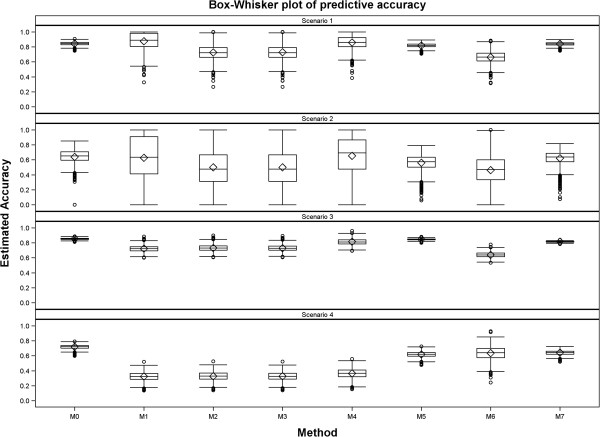
Box Whisker plot for predictive accuracy (estimates less than 0 were set to 0 whereas estimates greater than 1 were set to 1) for all the seven methods in each of the four scenarios.

**Figure 2:**
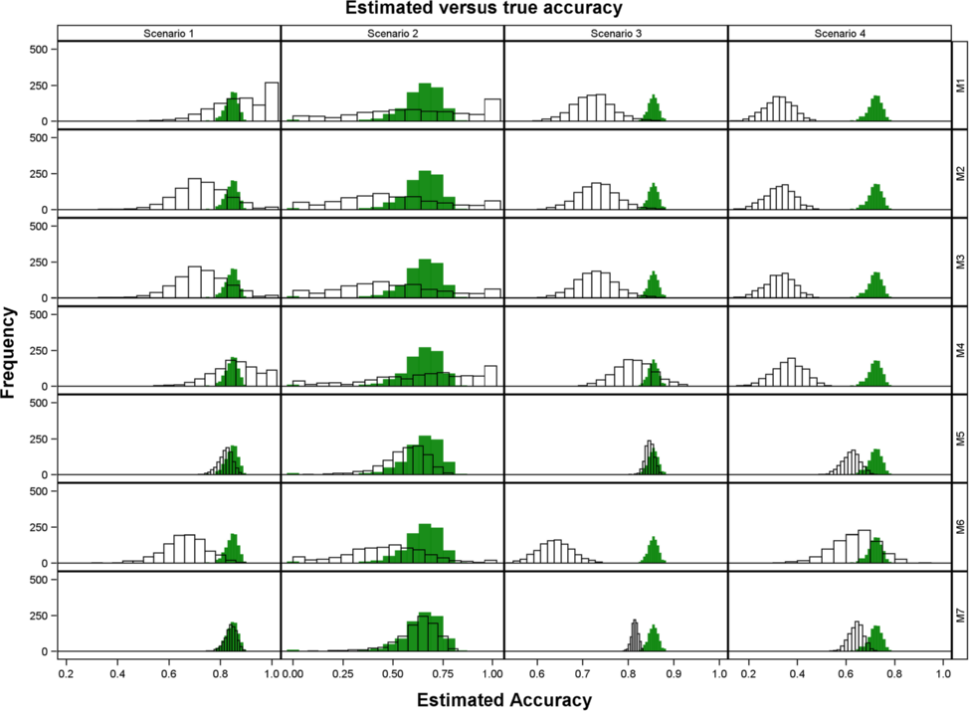
Frequency histograms for the true accuracy versus the estimated predictive accuracy (estimates less than 0 were set to 0 whereas estimates greater than 1 were set to 1) for all the seven methods in each of the four scenarios.

**Figure 3:**
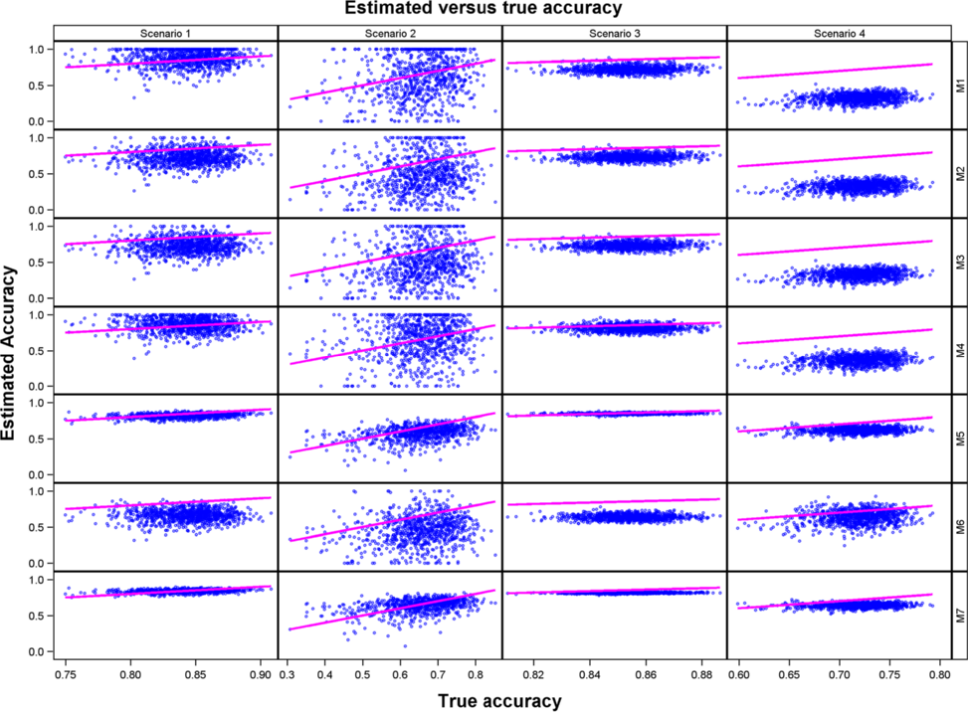
**Scatter plots of estimated predictive accuracy against the true simulated accuracy (estimates less than 0 were set to 0 whereas estimates greater than 1 were set to 1) for all the methods in each scenario.** The 1:1 (*y* = *x*) line is superimposed for comparison.

For each simulated dataset we computed the “true” correlation $r_{g,\hat{g}}$ (accuracy) as the correlation between the predicted and the simulated true breeding values and compared this with estimates of the same correlation computed using each of the seven different methods. To compare the true correlation $r_{g,\hat{g}}$ with its seven estimates ${\hat{r}}_{g,\hat{g}}$ we computed the mean squared deviation (MSD) of each estimate from the true correlation for each simulated dataset and method combination

(38) $$\text{MSD}=\sum_{j=1}^{N}\frac{{\left({\hat{r}}_{g,\hat{g},j}-r_{g,\hat{g},j}\right)}^{2}}{N},$$

where *N* is the total number of the simulated datasets and *j=* (1, 2, …, *N*) denotes the *j-*th simulated dataset. We recall here that MSD integrates information on (*i*) the correlation between the predicted and the true accuracy, (*ii*) the slope of the regression of the predicted against the true accuracy and (*iii*) the bias between the predicted and the true accuracy [26]. Nevertheless, the correlation and bias between the predicted and the simulated true accuracies are displayed or can readily be inferred from Figures 1, 2 and 3 and Additional file 3: Figures S1-S3 in the supplementary materials. Further, we calculated descriptive statistics for the true correlation and all its seven estimates. We also compared the estimated predictive accuracies between pairs of the seven methods.

For each scenario we compared the simulated true and the estimated predictive accuracies for the seven methods using *t*-tests (α = 5%) adjusted for multiplicity using simulation adjustment. The *t*-tests were derived from a mixed model with fixed effects for method and scenario and their interaction and a random effect for simulation replicates nested within scenarios [6].

## Results

### Heritability

Heritability was estimated by Methods 1 to 5 only. The estimated heritability was closer to its true simulated value for Methods 2, 3 and 5 than for Methods 1 and 4 in terms of its minimum, maximum, mean, standard deviation and mean squared deviation for all the four scenarios. All the five methods (Methods 1 to 5) underestimate the minimum, maximum and the mean true heritability in all the scenarios. Method 5 produced estimates closest to the true heritability for all the four scenarios. Across scenarios based on the same data set, the estimated heritability tended to be closer to its true value in Scenario 1 than in 2 and in Scenario 3 than in 4 (Table 5), implying that reducing the genetic variance by a factor of 10 in scenarios 2 and 4 reduced the accuracy of estimated heritability.

### Predictive accuracy

In general, all the seven methods produced reasonable estimates of predictive accuracy across all the four scenarios. The estimated predictive accuracy was more precise for Scenarios 3 and 4, based on the large dataset, than for Scenarios 1 and 2, for all the seven methods (Table 6, Figures 1, 2 and 3). Reducing the genetic variance by a factor of 10 while leaving all the other variance components unchanged degrades the precision of the estimated predictive accuracy more for the smaller of the two datasets (Table 6, Figures 1, 2 and 3 and Additional file 3: Figures S1-S4). Methods 5 and 7 were the best overall and gave the least biased and most precise estimates of predictive accuracy, most notably for Scenarios 1, 3 and 4 (Table 6 and Figures 1, 2 and 3). Even so, Method 5 tended to be more precise than Method 7 for both the scenarios (3 and 4) based on the larger dataset. All estimates of predictive accuracy for Methods 5 and 7 were between 0 and 1 for all scenarios. Also, the models for Methods 5 and 7 converged for all the 1000 simulated datasets in all the scenarios (Tables 5 and 6, Additional file 3: Figures S1-S2). Unlike Methods 5 and 7, the performances of the other methods were more varied across scenarios. In particular, there was a greater tendency for the estimated predictive accuracy to exceed one (“overshooting”) or to be smaller than zero (“undershooting”) and a higher frequency of failure of the algorithms used to fit the RR-BLUP models to converge (Additional file 3: Table S1, Additional file 3: Figures S1-S8).

For Scenario 1 overshooting was most frequent and severe (greater in magnitude) for Method 1 (21.8%, *n*=1000, range 1.0004-1.8960) followed by Methods 4 (6.8%, range 1.0015-1.1917), 2 (1.5%, range 1.0022-1.4152) and 3 (1.5%, range 1.0018-1.4153). In contrast, all estimates of predictive accuracy for Methods 5, 6 and 7 fell between 0 and 1. Moreover, all the seven models converged for all the 1000 simulated datasets (Table 6 and Additional file 3: Table S1).

Compared to Scenario 1, overshooting and undershooting were more common for Methods 1, 2, 3, 4 and 6 in Scenario 2. Generally, overshooting was more serious (greater in absolute magnitude) than undershooting, particularly for Methods 1 (*n*=164 datasets), 2 (*n*=65), 3 (*n*=65) and 4 (*n*=113) (Table 6, Additional file 3: Figures S1, S4 and S6). The problem of overshooting or undershooting did not occur for any method in Scenario 3. Although the problem of overshooting still persisted for Methods 1 (*n*=95), 2 (*n*=104), 3 (*n*=87) and 4 (*n*=163) in Scenario 4, these methods had three further drawbacks in Scenario 4. The first was the breakdown of the method for computing predictive accuracy because estimated heritability was zero, most especially for Methods 1 (*n*=27) and 2 (*n*=27). The second was that the genetic variance estimate was zero for Method 3 (*n*=27). The third was the failure of the mixed model used to compute predictive ability required by Methods 1, 2, 3, 4 and 6 to converge (*n*=18). As a result, predictive accuracy could not be computed for 45 datasets for each of the Methods 1, 2 and 3 in Scenario 4. Undershooting was rather rare by comparison and was noted only for Method 3 (*n*=17) in Scenario 4 (Additional file 3: Figures S2, S4 and S8). Aside from overshooting and undershooting, deviation of the predictive accuracy from its expected values was also caused by overestimation and underestimation. Considering only the values of predictive accuracy between 0 and 1, the seven methods tended to underestimate the true accuracy across all the four scenarios. Underestimation tended to be more severe for Methods 1, 2, 3, 4 and 6 than Methods 5 and 7. This was most evident in Scenario 4. By comparison, overestimation was far less common (Table 6, Figures 2 and 3, Additional file 3: Figure S2-S4).

For 11 datasets from Scenario 2 the estimated true accuracy was smaller than zero because the estimated variances of the BLUPs were zero and the estimated genetic variances were nearly zero. We expect plant breeders to discard genotypes for which the estimated genetic variance is nearly zero in making selection decisions in real applications. Consequently, we excluded the 11 simulated datasets with negative true accuracies from all the comparisons to mimic what plant breeders do in practice. There was therefore no estimated true accuracy to compare with the corresponding estimated predictive accuracy for all the seven methods. Similarly excluded from all the comparisons were four further datasets in Scenario 2 for which the mixed models for estimating the true accuracy did not converge.

**Figure 4:**
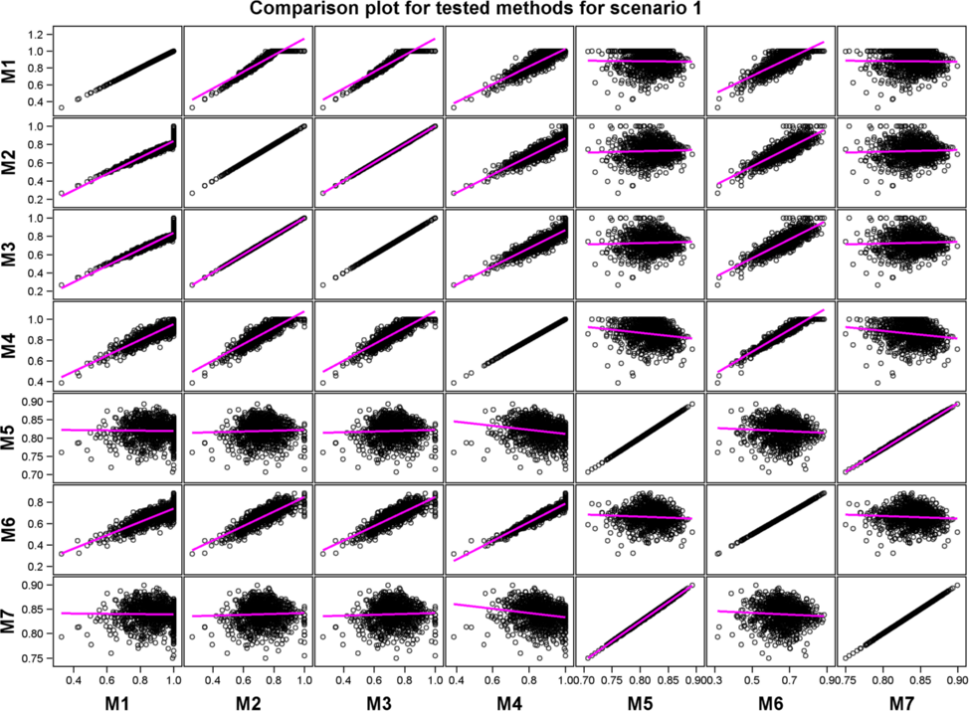
Scatter plots comparing all the estimated predictive accuracies for pairs of the seven tested methods for Scenario 1.

**Figure 5:**
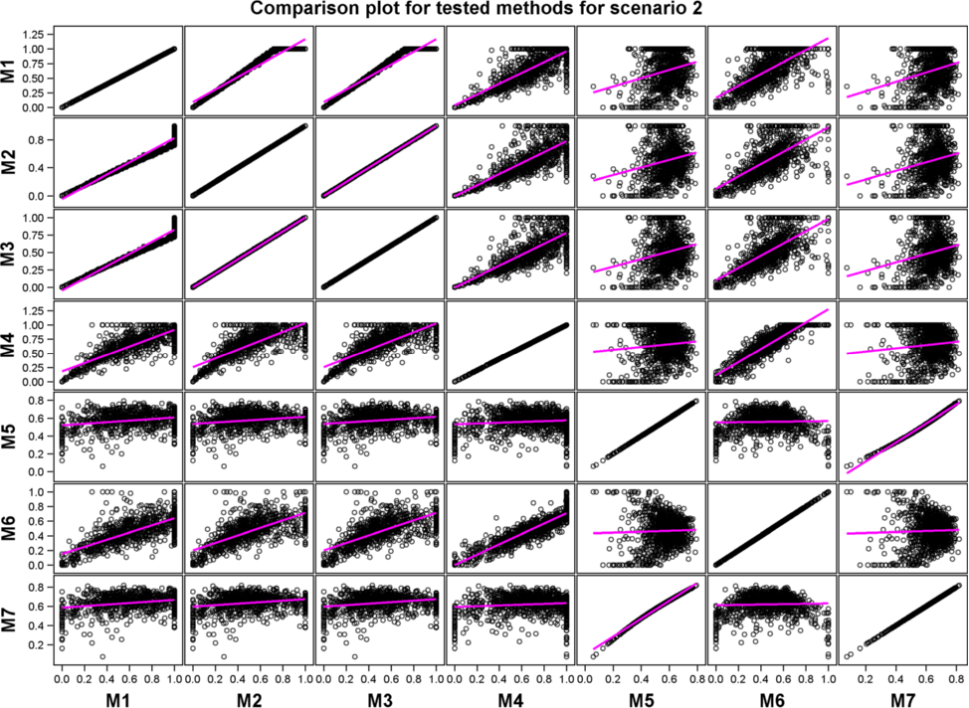
Scatter plots comparing all the estimated predictive accuracies for pairs of the seven tested methods for Scenario 2.

**Figure 6:**
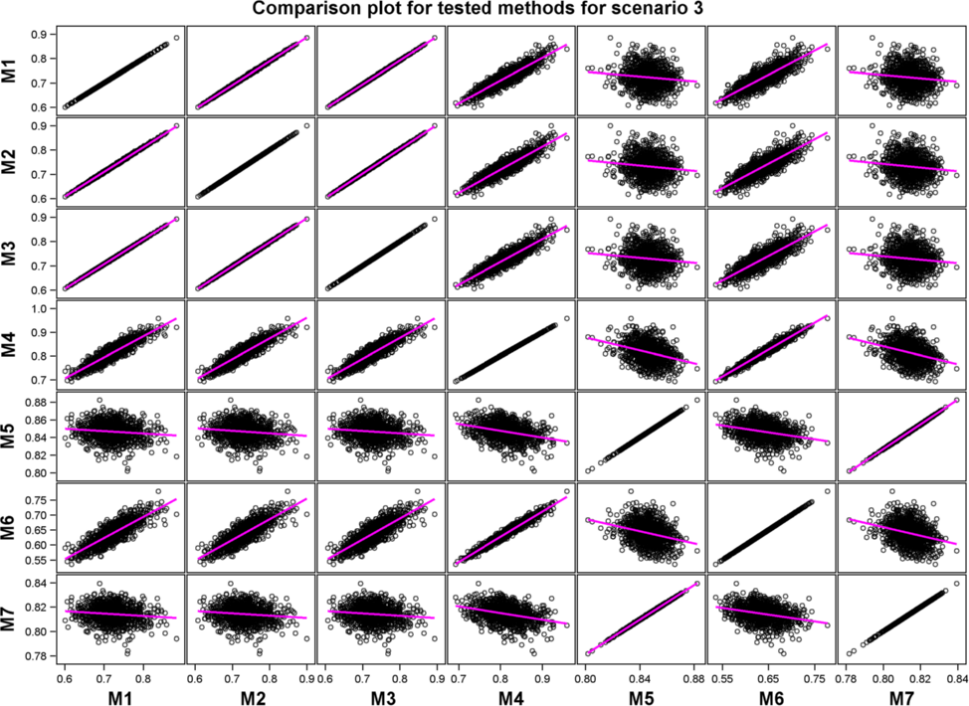
Scatter plots comparing all the estimated predictive accuracies for pairs of the seven tested methods for Scenario 3.

**Figure 7:**
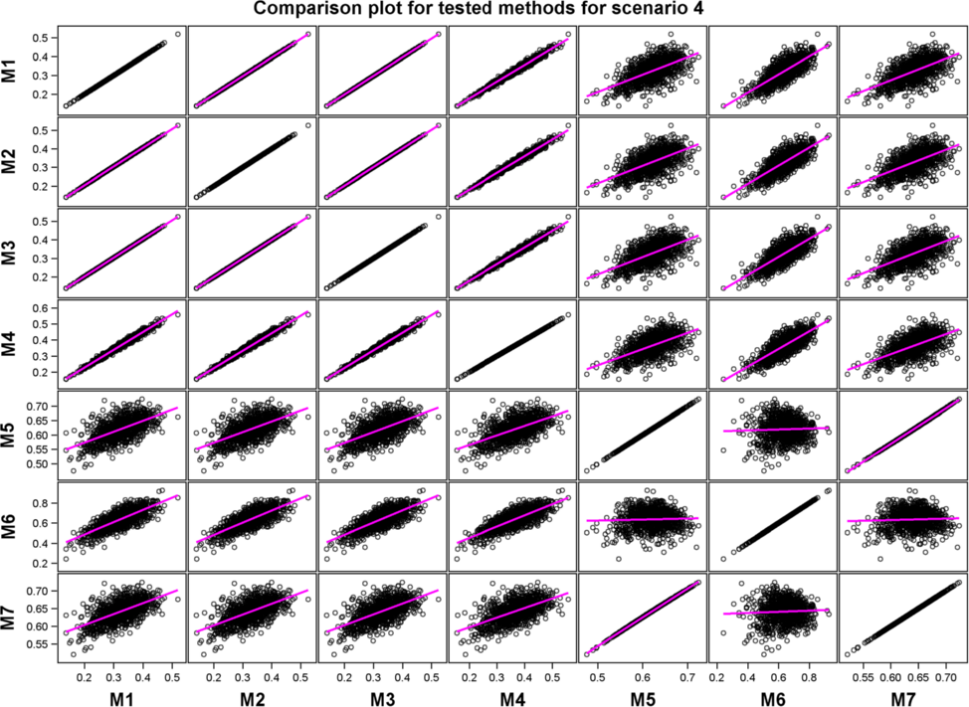
Scatter plots comparing all the estimated predictive accuracies for pairs of the seven tested methods for Scenario 4.

A comparison of the performances of the methods showed that methods with similar performances clustered into two distinct groups in each of the four scenarios. One of the two groups identified by regressing the estimated predictive accuracies for each pair of the seven methods on each other comprised Methods 1, 2, 3, 4, and 6 whereas the other consisted of Methods 5 and 7 (Table 6 and Additional file 3: Table S2, Figures 4, 5, 6 and 7 and Additional file 3: Figures S5-S8). Results of the *t*-tests reaffirmed this general pattern besides showing that the estimated true accuracies for Methods 5 and 7 are generally closer to the true predictive accuracy than the estimates for the other methods, especially for the two scenarios based on the large data set (Table 6 and Additional file 3: Table S1).

We expected the predictive accuracies of pairs of methods that accurately estimate the true predictive accuracy to be positively and not negatively correlated with each other. However, correlations between estimated predictive accuracies for some pairs of the seven methods were, surprisingly, negative even though the predictive accuracies for the individual methods were themselves high and positive (Table 7 and Additional file 3: Table S2, Figures 4, 5, 6 and 7 and Additional file 3: Figure S5-S8). This was the case, for example, for Method 4 versus 5 and 7 in Scenario 1, and for Method 5 versus 6 in Scenarios 2 and 3. This is due to the fact that some Methods (e.g., 5 and 7) tended to produce large values of predictive accuracy while others (e.g., Methods 4 and 6) tended to produce small values when the estimated genetic variance was very small because of the way the genetic variance enters the denominators of the estimators used by these methods to compute predictive accuracy.

**Table 7:** Correlation between predictive accuracies (estimates less than 0 were set to 0 whereas estimates greater than 1 were set to 1) for pairs of the seven methods by scenario

**Scenario**	**Method**	**M1**	**M2**	**M3**	**M4**	**M5**	**M6**	**M7**
1	M1	1.00						
	M2	0.94	1.00					
	M3	0.94	1.00	1.00				
	M4	0.90	0.89	0.89	1.00			
	M5	-0.02	0.04	0.04	-0.18	1.00		
	M6	0.81	0.84	0.84	0.96	-0.06	1.00	
	M7	-0.02	0.04	0.04	-0.18	1.00	-0.07	1.00
2	M1	1.00						
	M2	0.97	1.00					
	M3	0.97	1.00	1.00				
	M4	0.82	0.78	0.78	1.00			
	M5	0.21	0.17	0.17	0.04	1.00		
	M6	0.70	0.67	0.67	0.91	-0.02	1.00	
	M7	0.26	0.22	0.22	0.11	0.95	0.03	1.00
3	M1	1.00						
	M2	1.00	1.00					
	M3	1.00	1.00	1.00				
	M4	0.91	0.91	0.91	1.00			
	M5	-0.11	-0.12	-0.12	-0.32	1.00		
	M6	0.83	0.83	0.83	0.98	-0.28	1.00	
	M7	-0.11	-0.12	-0.12	-0.32	1.00	-0.28	1.00
4	M1	1.00						
	M2	1.00	1.00					
	M3	1.00	1.00	1.00				
	M4	0.99	0.99	0.99	1.00			
	M5	0.59	0.59	0.59	0.58	1.00		
	M6	0.77	0.77	0.77	0.79	0.04	1.00	
	M7	0.59	0.59	0.59	0.58	1.00	0.05	1.00

The number of simulated datasets out of a possible total of 1000 for which estimates of predictive were available for each pair of methods was taken as the minimum for the pair.

## Discussion

### Heritability

One possible definition of heritability [27] is based on the squared correlation between “genotype” and “phenotype”. In our current work, we use various *ad hoc* measures of heritability. The one proposed by [18] assesses the squared correlations between BLUPs of genotypic values and the true genotypic values using Method 3. Another measure, proposed by [16], considers the correlation between adjusted means (BLUEs of genotypic values) and the true genotypic values using Method 2. So, one may argue that these different measures use different definitions of “the phenotype”.

In this paper, we use estimates of heritability to compute “predictive accuracy” as $r_{\hat{g},p}/H$, where $r_{\hat{g},p}$ is the correlation between genomic selection estimators of the true breeding values *g* and the “phenotypic values” in the validation set and *H* is the square root of heritability. In this application, the “phenotypes” are adjusted means (BLUEs), so estimators of the square root of heritability *H* that take BLUEs to be the phenotype are appropriate.

It is important to note that $r_{\hat{g},p}/H$ is itself an estimator of the square root of heritability if we take the RR-BLUPs as the “phenotypes”. An important property of the procedures we consider (this provides estimators of *H* by taking RR-BLUPs to be the phenotypes) is that $r_{\hat{g},p}$ is obtained from cross-validation. Alternatively, we also estimate heritability *H*^2^ (or *H*) without cross-validation using similar ideas to those used for the *ad hoc* measures of heritability *H*^2^ and for the “direct method” (Method 5). The quantity $H_{m5}^{2}$ (24) provides an alternative estimator of heritability when the RR-BLUP of the genetic variance (*g*) is considered as the “phenotype”. This estimator ($H_{m5}^{2}$) was the most accurate of the five we used to compute heritability. But in our cross-validations, the phenotypes are always the adjusted means. So, we just use (24) as an alternative estimator of predictive accuracy.

The strikingly poor performances of Methods 1 to 3 as indicated by all the estimated mean heritabilities falling below the minimum of the true heritability may seem surprising at first sight but in the case of Methods 1 to 3 may reflect the fact that these three methods assume that genotypes are uncorrelated. If this is true then we would expect the performance of these methods to improve considerably if the true heritability used as the benchmark were also estimated using a model that assumes uncorrelated genotypes. To test this expectation, we re-calculated the true heritability assuming that the genotypes are uncorrelated for each of the four scenarios by setting all the covariances in the variance-covariance matrix of genotypes to zero. Using the new benchmark led, as expected, to a much better agreement between the new true heritability and its estimates by Methods 1 to 3 (Additional file 3: Table S2). This demonstrates compellingly that Methods 1 to 3 are all reasonably good at estimating heritability when genotypes are not correlated but are severely biased downwards when they are. The downward bias implies, furthermore, that Methods 1 to 3 are not suited for estimating heritability used to divide predictive ability to obtain predictive accuracy in genomic prediction for which genotypes are typically assumed to be correlated. By contrast, Method 5 that assumes correlated genotypes performs much better at estimating heritability even though the estimated heritability is merely a by-product and not needed for estimating predictive accuracy.

Method 4 differs from Methods 1 to 3 in assuming that the genotypes are correlated, unlike the latter methods that assume that genotypes are independent. This suggest that there must be yet another reason that the estimated mean heritability for Method 4 falls below the minimum expected true heritability estimated assuming that the genotypes are correlated. We can think of two plausible explanations for this discrepancy both of which apply equally to all Methods 1 to 4. The first is that all the heritability estimators for Methods 1 to 4 are constructed as ratios of the genetic and the phenotypic variances such that the greater is the phenotypic variance the smaller is the estimated heritability, whereas we defined the true heritability in terms of the squared correlation between true and estimated genotypic effect. The second relates to the observation that despite its poor estimates of heritability relative to those for Methods 1 to 3, the estimated predictive accuracy for Method 4 is generally closer to the estimated true accuracy than the estimates for Methods 1 to 3. This is quite intriguing because Methods 1 to 4 calculate predictive accuracy as the ratio of predictive ability to heritability. Since all the four methods use the exact same values of predictive ability and since Method 4 yields somewhat poorer estimates of heritability than Methods 1 to 3, we would not logically expect Method 4 to produce better estimates of predictive accuracy. That it actually did suggests that the reliability of approximating predictive accuracy by dividing predictive ability by the square root of heritability is questionable, at the very least for the specific configurations of the phenotypic and genotypic variances we used in our simulation scenarios.

### Predictive accuracy

We compared the performances of seven methods for estimating predictive accuracy in genomic selection using 1000 datasets simulated according to an alpha-design for each of four scenarios based on genetic and residual variance estimates calculated from two real datasets. The results show that, of the seven methods, a new proposed method (Method 5) and a method which is well established in animal breeding programs (Method 7, [18]), consistently gave the least biased, most precise and stable estimates of predictive accuracy across all the four scenarios. Method 5 was at least as good as or better than Method 7 for estimating predictive accuracy. The other methods performed somewhat inconsistently across scenarios and suffered varying degrees of overshooting, undershooting and convergence problems. All the methods were more likely to underestimate than overestimate the true predictive accuracy when only datasets for which the estimated predictive accuracies fell between 0 and 1 were considered. The 0–1 truncation of the estimated predictive accuracy reflects the fact that we should not use the ratio of predictive ability to heritability ($r_{\hat{g},p}/H$) in practice if it does not fall within the interval [0, 1].

In summary, Methods 5 and 7 had the best performance followed by Method 4. Method 6 was the third best whereas Methods 1, 2 and 3 had rather similar and the worst performance. Methods 5 and 7 had rather similar performance in all scenarios despite the theoretical expectation that Method 5 should do better than Method 7 for the scenarios with small sample size. This expectation arises from the fact that whereas Method 7 assumes that the focal genotypes are derived from an infinite target population, Method 5 assumes that the sampled genotypes arise from a finite population. Consequently, the two methods may be expected to perform well for large sample sizes and Method 5 to perform better than Method 7 in small sample situations. The similar performance of Methods 5 and 7 is therefore tentative and its generality will be explored for a wider range of sample sizes in a sequel to this paper focusing on the influence of sample size on the predictive performance of the seven methods.

Although their empirical performances in the simulations were often reasonable, Methods 1, 2 and 3 involve dividing two quantities computed using two different models with conflicting assumptions. Specifically, they involve dividing predictive ability computed from an RR-BLUP model, assuming that genotypes are correlated, by the square root of heritability computed from a model assuming that genotypes are uncorrelated. The computation of predictive ability using the model assuming that the genotypes are correlated, when heritability is computed assuming uncorrelated genotypes, would seem unavoidable when using RR-BLUP. This is because genomic breeding values cannot be predicted using RR-BLUP if the genotypes are assumed to be independent. This theoretical inconsistency undermined the performance of these three methods in several instances when the genetic variance estimated assuming uncorrelated genotypes was zero or nearly zero. This rendered predictive accuracy inestimable for Methods 1, 2 and 3 in these cases.

Despite the inferior performance of Methods 1 to 3, linked to the inconsistency in the definitions of their numerators and denominators, relative to the methods that estimate predictive accuracy directly, this theoretical inconsistency has not deterred plant breeders from using these approaches. In fact, Methods 1 to 3 are the most frequently used by plant breeders. These three methods are not very similar by construction, despite the similarity of their performance. Hence, the similar performance could not necessarily have been anticipated a priori. Accordingly, by studying the properties of these methods alongside those of the other contending methods, we have ascertained whether and when their theoretical inconsistency may lead to inferior performance. Our findings suggest that these methods should be used with care, especially when the genetic variance is very small, so that predictive accuracy is likely to be either inestimable or overestimated.

Although rare and probably relatively simple, there are instances in which the theoretical inconsistency is immaterial. For example, an independent model can be obtained in such simple special cases as a doubled haploid population, resulting from a single cross [2], if the dependent model is a conditional model that considers genetic variance-covariance conditioning on the marker genotypes whereas the marker genotypes are taken as random variables. In complex pedigrees, however, the unconditional model will also involve correlations, so that the impact of the theoretical inconsistency is more likely to be consequential.

We emphasize also that while our simulation results hold for the RR-BLUP model, their applicability to other models than RR-BLUP remains to be investigated.

### Influence of the size of genetic variance

The size of the estimated genetic variance and hence heritability exerted the strongest influence on the variation in estimates of predictive accuracy. The estimated predictive accuracy was closer to its true value for all the methods in Scenarios 1 and 3 than in Scenarios 2 and 4 for all the 1000 datasets, because the simulated genetic variances for Scenarios 1 and 3 were 10 times larger than those for Scenarios 2 and 4. When the simulated genetic variance was small (Scenarios 2 and 4), there was a higher likelihood of obtaining extremely high values of estimated genetic variances than when a higher genetic variance was simulated (Scenarios 1 and 3). Methods 1, 2 and 3 were the most sensitive to variation in the estimated genetic variance because they all divide predictive ability by the square root of heritability to obtain predictive accuracy and hence break down when genetic variance estimate is zero, since the estimated heritability is then also zero. For Method 3, in particular, predictive accuracy becomes infinitely large when the estimated genetic variance is zero and extremely large when this variance is very small. Since Method 4 also computes predictive accuracy in the same way as Methods 1, 2 and 3 do, it also breaks down when the estimated genetic variance and hence estimated heritability is zero.

### Influence of the number of genotypes

Increasing the number of genotypes, for example from 177 for Scenarios 1 and 2, to 698 for Scenarios 3 and 4, increased the time required to compute predictive accuracy by all the seven methods from a few minutes to several days, most notably for the methods that require cross-validation (Methods 1, 2, 3, 4 and 6).

### Simplicity of implementation of methods

Methods 1, 2, 3, 4 and 6 that use cross-validation are computationally much more intensive to implement than Methods 5 and 7 that do not involve cross-validation. As a result, Methods 5 and 7 are the simplest and computationally most efficient to implement of the seven methods. This argues for their routine use in assessing predictive accuracy in genomic selection studies.

Considering only estimated correlations between zero and one, Methods 4 and 6 gave the best estimates of predictive accuracy among the five methods that use cross-validation, followed by Methods 3, 2 and 1, in that order.

### Design considerations

We considered a single trial laid out as an alpha-design in a single location for simplicity. Hence, a more extensive simulation with more trials, locations and trial designs would be required to establish the generality of our results. Further, we considered only two data sets with different numbers of genotypes (177 and 698) and markers (275 and 11646). However, variation in the number of genotypes and markers probably also affects the estimated predictive accuracy and thus also merit further investigation. Some breeders compute heritability using methods assuming that the datasets are balanced and that the genotypes are independent. Four of the seven methods (Methods 4, 5, 6 and 7) relax these restrictive assumptions by allowing for both balanced and unbalanced datasets as well as for independent and correlated genotypes.

## Conclusions

Methods 5 and 7 were the most computationally efficient to implement and gave consistently the most accurate, robust and stable estimates of predictive accuracy of the seven methods across all the four scenarios. These properties argue for their routine use in assessing predictive accuracy in genomic selection studies. Among the five methods that use cross-validation, Methods 4 and 6 performed better than Methods 1, 2 and 3 but were clearly inferior to Methods 5 and 7. Both the genetic variance and the number of genotypes exerted strong influences on predictive accuracy. Thus, predictive accuracy was higher for the larger data set. Furthermore, reducing the genetic variance degraded predictive accuracy much more for the smaller of the two data sets. We are investigating the influences of genetic variance and the number of genotypes on predictive accuracy in genomic selection in greater detail in a sequel to this paper.

## Abbreviations

GS: Genomic selection; RR-BLUP: Ridge-regression BLUP; BLUE: Best linear unbiased estimation; BLUP: Best linear unbiased prediction; REML: Restricted maximum likelihood; SNP: Single nucleotide polymorphism; CV: Cross-validation; H2: Heritability; MME: Mixed model equations;

$$\hat{g}$$:

BLUP of the genetic effects *g*; p: the BLUE of genotype means (“phenotypes”); S: Sample standard deviation; $s_{g}^{2}$: Sample variance of the true genetic breeding values *g*; $s_{\hat{g}}^{2}$: Sample variance of the predicted breeding value; $s_{p}^{2}$: Phenotypic sample variance; σ: Population standard deviation; $\sigma_{g}^{2}$: Population variance of the true genetic breeding values *g*; r: Sample correlation; $r_{g,\hat{g}}$: Sample correlation between the true and the predicted breeding values; $r_{\hat{g},p}$: Sample correlation between the BLUP of *g* and the observed “phenotypes” *p*; ρ: Population correlation; ρg,p: Population correlation between the true genetic breeding values *g* and the observed “phenotypes” *p*; $s_{g,\hat{g}}$: Sample covariance between the true and predicted breeding values.

## Competing interests

The authors declare that they have no competing interests.

## Authors’ contributions

SBOE and JOO participated in the design of the study, conducting the simulations, interpreting the results, drafting and editing the manuscript. TSS participated in conducting the simulations. CK and MO conceived and designed the field trials and supervised the collection of the Synbreed data set. AG conceived and designed the field trials and supervised the collection of the AgReliant data set. HPP conceived the study, participated in its design, writing and editing the manuscript and oversaw the project. All authors read and approved the final manuscript.

## Supplementary Material

Additional file 1 How to compute the average variance of a difference from the variance-covariance matrix of adjusted means. Click here for file

Additional file 2 Three special cases for the new Method 4. Click here for file

Additional file 3 Descriptive statistics for predictive accuracy by scenario (Table S1), Descriptive statistics for the estimated true heritability assuming that genotypes are not correlated for each of the four scenarios (Table S2), Box Whisker plot of all the predictive accuracies for scenario 2 (Figure S1), Box Whisker plot of all the predictive accuracies for scenario 4 (Figure S2), Frequency histograms for the true (green) versus the estimated (white) predictive accuracy for all the seven methods and four scenarios (Figure S3), Scatter plots of estimated predictive accuracy against the true accuracy for all the seven methods and four scenarios (Figure S4), Scatter plots comparing the estimated predictive accuracies for pairs of the seven tested methods for scenario 1 (Figure S5), Scatter plots comparing the estimated predictive accuracies for pairs of the seven tested methods for scenario 2 (Figure S6), Scatter plots comparing the estimated predictive accuracies for pairs of the seven tested methods for scenario 3 (Figure S7) and Scatter plots comparing the estimated predictive accuracies for pairs of the seven tested methods for scenario 4 (Figure S8). Click here for file

Additional file 4 SAS (version 9.3) code used to simulate phenotypic data and implement all the seven methods. Click here for file

## References

[B1] Meuwissen THE Hayes BJ Goddard ME Prediction of total genetic value using genome-wide dense marker map Genetics 2001 14 1819 1829 11290733 10.1093/genetics/157.4.1819 PMC1461589

[B2] Piepho HP Ridge regression and extensions for genomewide selection in maize Crop Sci 2009 14 1165 1176 10.2135/cropsci2008.10.0595

[B3] Whittaker JC Thomson R Denham MC Marker-assisted selection using ridge regression Genetic Research 2000 14 249 252 10.1017/S0016672399004462 10816982

[B4] Bernardo R Yu J Prospects for genomewide selection for quantitative traits in maize Crop Sci 2007 14 1082 1090 10.2135/cropsci2006.11.0690

[B5] Goddard ME Hayes BJ Genomic selection J Anim Breed Genet 2007 14 323 330 10.1111/j.1439-0388.2007.00702.x 18076469

[B6] Schulz-Streeck T Ogutu JO Karaman Z Knaak C Piepho HP Genomic selection using multiple populations Crop Sci 2012 14 2453 2461 10.2135/cropsci2012.03.0160

[B7] Visscher PM Hill WG Wray NR Heritability in the genomics era-concepts and misconceptions Nat Rev Genet 2008 14 255 266 18319743 10.1038/nrg2322

[B8] Dekkers JCM Prediction of response to marker-assisted and genomic selection using selection index theory J Anim Breed Genet 2007 14 331 341 10.1111/j.1439-0388.2007.00701.x 18076470

[B9] Habier D Tetens J Seefried FR Lichtner P Thaller G The impact of genetic relationship information on genomic breeding values in German Holstein cattle Genet Sel Evol 2010 14 5 20170500 10.1186/1297-9686-42-5 PMC2838754

[B10] Schulz-Streeck T Ogutu JO Piepho H-P Comparisons of single-stage and two-stage approaches to genomic selection Theor Appl Genet 2013 14 69 82 10.1007/s00122-012-1960-1 22903736

[B11] Petersen RG Agricultural Field Experiments/Design and analysis 1994 New York: Marcel Dekker

[B12] Albrecht T Wimmer V Auinger HJ Erbe M Knaak C Ouzunova M Simianer H Schön CC Genomic-based prediction of testcross values in maize Theor Appl Genet 2011 14 339 350 10.1007/s00122-011-1587-7 21505832

[B13] SAS Institute Inc SAS Institute Inc 2013 NC: Cary

[B14] Legarra A Robert-Granie C Manfredi E Elsen J Performance of genomic selection in mice Genetics 2008 14 611 618 10.1534/genetics.108.088575 18757934 PMC2535710

[B15] Hastie T Tibshirani R Friedman J The Elements of Statistical Learning: Data Mining, Inference and Prediction 2009 2 New York: Springer

[B16] Piepho HP Möhring J Computing heritability and selection response from unbalanced plant breeding trials Genetics 2007 14 1881 1888 10.1534/genetics.107.074229 18039886 PMC2147938

[B17] Gonçalves E Carrasquinho I St Aubyn A Martins A Broad-sense heritability in mixed models for grapevine initial selection trials Euphytica 2013 14 379 391 10.1007/s10681-012-0787-9

[B18] Cullis BR Smith A Coombes N On the design of early generation variety trials with correlated data J Agr Biol Envir St 2006 14 381 393 10.1198/108571106X154443

[B19] Johnson NL Kotz S Kemp AW Univariate Discrete Distribution 1993 2 New York: Wiley

[B20] Mrode RA Thompson R Linear Models for the Prediction of Animal Breeding Values 2005 2 Wallingford: UK

[B21] Henderson CR Comparison of alternative sire evaluation methods J Anim Sci 1975 14 760 770

[B22] VanRaden PM Efficient methods to compute genomic predictions J Dairy Sci 2008 14 4414 4423 10.3168/jds.2007-0980 18946147

[B23] McLean RA Sanders WL Stroup WW A unified approach to mixed linear models Am Stat 1991 14 54 63

[B24] Henderson CR Best linear unbiased prediction of breeding values not in the model for records J Dairy Sci 1977 14 783 787 10.3168/jds.S0022-0302(77)83935-0

[B25] Searle SR Casella G McCulloch CE Variance Components 1992 New York: Wiley

[B26] Gauch HG Jr Gene Hwang JT Fick GW Model evaluation by comparison of model-based predictions and measured values Agron J 2003 14 1442 1446 10.2134/agronj2003.1442

[B27] Holland JB Nyquist WE Cervantes-Martinez CT Estimating and interpreting heritability for plant breeding: an update J Plant Breed 2003 14 9 112

