# Supplementary material for: Evaluation of approaches for estimating the accuracy of genomic prediction in plant breeding
Source: BMC Genomics. 2013 Dec 6;14:860. doi: 10.1186/1471-2164-14-860 (PMC3879103; doi:10.1186/1471-2164-14-860)
Supplement: Additional file 1 — How to compute the average variance of a difference from the variance-covariance matrix of adjusted means. [file 1471-2164-14-860-S1.doc]

**How to compute the average variance of a difference from the variance-covariance matrix of adjusted means**

Let $m$ be the vector of adjusted means and let $V_{m}$ be the estimate of $\mathrm{var} \left(m\right)$, the variance-covariance matrix of adjusted means. Let $D$ be a contrast matrix with $n$ columns and $n\left(n-1\right)/2$ rows that generates all pairwise differences. For example, when $n=4$ we have

$D=\left(\begin{matrix} 1 & -1 & 0 & 0 \\ 1 & 0 & -1 & 0 \\ 1 & 0 & 0 & -1 \\ 0 & 1 & -1 & 0 \\ 0 & 1 & 0 & -1 \\ 0 & 0 & 1 & -1 \end{matrix}\right)$.

The variance-covariance matrix of all pairwise differences, $d=Dm$ is given by

$V_{d}=DV_{m}D^{T}$.

This matrix has variances of all pairwise differences along the diagonal. So the mean variance of a difference is

$\bar{v}_{d}=\frac{2}{n\left(n-1\right)}trace\left(DV_{m}D^{T}\right)$.

To evaluate the trace, it is helpful to exploit the fact that

$trace\left(DV_{m}D^{T}\right)=trace\left(V_{m}D^{T}D\right)$.

Moreover, we have $D^{T}D=nI_{n}-1_{n}1_{n}^{T}$, where $I_{n}$ is the *n*-dimensional identity matrix and $1_{n}$ is an *n*-vector of ones. Thus,

$trace\left(DV_{m}D^{T}\right)=trace\left(V_{m}D^{T}D\right)=n\times trace\left(V_{m}\right)-trace\left(V_{m}1_{n}1_{n}^{T}\right)=n\times trace\left(V_{m}\right)-1_{n}V_{m}1_{n}^{T}$.

The second quantity on the right-hand side of the above equation, $1_{n}V_{m}1_{n}^{T}$ is the sum of all the elements of $V_{m}$. The average variance of a difference can thus be computed as

$\bar{v}_{d}=\frac{2}{n\left(n-1\right)}\left[n\times trace\left(V_{m}\right)-1_{n}V_{m}1_{n}^{T}\right]$ [1].

**References**

1. de S. Bueno FJ S, Gilmour SG: **Planning Incomplete Block Experiments When Treatments Are Genetically Related**. *Biometrics* 2003, **59**: 375–381.
