# Supplementary material for: Evaluation of approaches for estimating the accuracy of genomic prediction in plant breeding
Source: BMC Genomics. 2013 Dec 6;14:860. doi: 10.1186/1471-2164-14-860 (PMC3879103; doi:10.1186/1471-2164-14-860)
Supplement: Additional file 2 — Three special cases for the new Method 4. [file 1471-2164-14-860-S2.doc]

**Three special cases for the new Method 4**

For illustration of the new Method 4, we consider three special cases. (1) Randomized complete block design with fixed blocks, and independent genotypes

$G=I_{n}s_{g}^{2}$, $R=I_{n}\sigma_{e}^{2}$,

$H_{m4}^{2}=\frac{E\left(s_{g}^{2}\right)}{E\left(s_{p}^{2}\right)}=\frac{s_{g}^{2}trace\left(P_{u}\right)}{s_{g}^{2}trace\left(P_{u}\right)+\sigma_{e}^{2}trace\left(P_{u}\right)}=\frac{s_{g}^{2}}{s_{g}^{2}+\sigma_{e}^{2}}$.

Note that $trace\left(P_{u}\right)=\frac{1}{n-1}trace\left(I_{n}-\frac{1}{n}J_{n}\right)=1$.

(2) Randomized complete block design with random blocks, and independent genotypes

$G=I_{n}\sigma_{g}^{2}$, $R=J_{n}\varphi+I_{n}\sigma_{e}^{2}$,

where $\varphi$ is a covariance resulting from the random block effects.

$H_{m4}^{2}=\frac{E\left(s_{g}^{2}\right)}{E\left(s_{p}^{2}\right)}=\frac{s_{g}^{2}trace\left(P_{u}\right)}{s_{g}^{2}trace\left(P_{u}\right)+\varphi trace\left(J_{n}P_{u}\right)+\sigma_{e}^{2}trace\left(P_{u}\right)}=\frac{s_{g}^{2}}{s_{g}^{2}+\sigma_{e}^{2}}$, because $J_{n}P_{u}=0$.

So, we are reproducing the standard formula in simple cases. But: this formula is entirely general; it also works for the model underlying RR-BLUP.

(3) Arbitrary design, and independent genotypes

$G=I_{n}\sigma_{g}^{2}$, $R$ arbitrary,

$H_{m4}^{2}=\frac{E\left(s_{g}^{2}\right)}{E\left(s_{p}^{2}\right)}=\frac{s_{g}^{2}trace\left(P_{u}\right)}{s_{g}^{2}trace\left(P_{u}\right)+trace\left(P_{u}R\right)}=\frac{s_{g}^{2}}{s_{g}^{2}+\frac{1}{2}\bar{v}}$,

where $\bar{v}$ is the variance of a difference (It is easily verified that $trace\left(P_{u}R\right)=\frac{1}{2}\bar{v}$). This is the *ad hoc* measure proposed in [1]. So, we have a very simple generalization of that approach to models with correlated genotypic effects.

**References**

1. Piepho HP, Möhring J: **Computing heritability and selection response from**

**unbalanced plant breeding trials**. *Genetics* 2007, **177**: 1881-1888.
