# Supplementary material for: Evaluation of approaches for estimating the accuracy of genomic prediction in plant breeding
Source: BMC Genomics. 2013 Dec 6;14:860. doi: 10.1186/1471-2164-14-860 (PMC3879103; doi:10.1186/1471-2164-14-860)
Supplement: Additional file 3 — Descriptive statistics for predictive accuracy by scenario (Table S1), Descriptive statistics for the estimated true heritability assuming that genotypes are not correlated for each of the four scenarios (Table S2), Box Whisker plot of all the predictive accuracies for scenario 2 (Figure S1), Box Whisker plot of all the predictive accuracies for scenario 4 (Figure S2), Frequency histograms for the true (green) versus the estimated (white) predictive accuracy for all the seven methods and four scenarios (Figure S3), Scatter plots of estimated predictive accuracy against the true accuracy for all the seven methods and four scenarios (Figure S4), Scatter plots comparing the estimated predictive accuracies for pairs of the seven tested methods for scenario 1 (Figure S5), Scatter plots comparing the estimated predictive accuracies for pairs of the seven tested methods for scenario 2 (Figure S6), Scatter plots comparing the estimated predictive accuracies for pairs of the seven tested methods for scenario 3 (Figure S7) and Scatter plots comparing the estimated predictive accuracies for pairs of the seven tested methods for scenario 4 (Figure S8). [file 1471-2164-14-860-S3.doc]

**Table S1**. Descriptive statistics for predictive accuracy by scenario. All methods except 5 and 7 use cross-validation. M0 is the correlation between the predicted and the true simulated breeding values used as the benchmark for assessing the estimated predictive accuracy. The number in parenthesis after the name of each method refers to equations in the text. The number of simulated datasets for each pair of methods was taken as the minimum for the pair (N).

|  |  | Methods | | | | | | | | |
| --- | --- | --- | --- | --- | --- | --- | --- | --- | --- | --- |
| Scenario | ^†^Statistic | M0 | ^*^M1(12) | | M2(13) | M3(14) | M4(18) | M5(20) | M6(22) | M7(32) |
|  |  | $r_{g,\hat{g}}$ |  | |  |  |  | $\hat{H}_{m5}$ |  | $\hat{\rho}_{m7}$ |
| 1 | N | 1000 | 1000 | | 1000 | 1000 | 1000 | 1000 | 1000 | 1000 |
|  | MIN | 0.750 | 0.327 | | 0.265 | 0.265 | 0.384 | 0.707 | 0.316 | 0.750 |
|  | MEAN | 0.843 | 0.899 | | 0.728 | 0.728 | 0.861 | 0.819 | 0.663 | 0.840 |
|  | MAX | 0.908 | 1.896 | | 1.415 | 1.415 | 1.192 | 0.893 | 0.884 | 0.899 |
|  | STD | 0.024 | 0.146 | | 0.107 | 0.107 | 0.098 | 0.028 | 0.084 | 0.023 |
|  | MSD | 0.000 | 0.025 | | 0.025 | 0.025 | 0.011 | 0.002 | 0.040 | 0.001 |
|  | Q1 | 0.829 | 0.807 | | 0.661 | 0.661 | 0.802 | 0.803 | 0.612 | 0.826 |
|  | Median | 0.846 | 0.890 | | 0.723 | 0.724 | 0.862 | 0.822 | 0.665 | 0.841 |
|  | Q3 | 0.860 | 0.983 | | 0.793 | 0.793 | 0.923 | 0.839 | 0.716 | 0.856 |
|  |  |  |  | |  |  |  |  |  |  |
| 2 | N | 839 | 839 | | 839 | 839 | 839 | 839 | 839 | 839 |
|  | MIN | 0.31 | | -0.24 | -0.17 | -0.17 | -0.87 | 0.06 | -0.83 | 0.08 |
|  | MEAN | 0.65 | 0.76 | | 0.56 | 0.56 | 0.66 | 0.57 | 0.46 | 0.64 |
|  | MAX | 0.85 | 9.76 | | 6.90 | 6.90 | 6.95 | 0.79 | 4.44 | 0.82 |
|  | STD | 0.09 | 0.70 | | 0.50 | 0.50 | 0.37 | 0.10 | 0.26 | 0.09 |
|  | MSD | 0.000 | 0.502 | | 0.257 | 0.257 | 0.141 | 0.018 | 0.108 | 0.011 |
|  | Q1 | 0.61 | 0.42 | | 0.31 | 0.31 | 0.47 | 0.53 | 0.33 | 0.59 |
|  | Median | 0.66 | 0.64 | | 0.48 | 0.48 | 0.68 | 0.58 | 0.47 | 0.65 |
|  | Q3 | 0.71 | 0.91 | | 0.67 | 0.67 | 0.85 | 0.64 | 0.59 | 0.70 |
|  |  |  |  | |  |  |  |  |  |  |
| 3 | N | 1000 | 1000 | | 1000 | 1000 | 1000 | 1000 | 1000 | 1000 |
|  | MIN | 0.81 | 0.60 | | 0.61 | 0.61 | 0.69 | 0.80 | 0.54 | 0.78 |
|  | MEAN | 0.85 | 0.72 | | 0.73 | 0.73 | 0.81 | 0.85 | 0.64 | 0.81 |
|  | MAX | 0.89 | 0.88 | | 0.90 | 0.89 | 0.96 | 0.88 | 0.78 | 0.84 |
|  | STD | 0.01 | 0.04 | | 0.04 | 0.04 | 0.04 | 0.01 | 0.04 | 0.01 |
|  | MSD | 0.0000 | 0.0193 | | 0.0169 | 0.0176 | 0.0036 | 0.0002 | 0.0477 | 0.0017 |
|  | Q1 | 0.85 | 0.69 | | 0.70 | 0.70 | 0.79 | 0.84 | 0.61 | 0.81 |
|  | Median | 0.85 | 0.72 | | 0.73 | 0.73 | 0.81 | 0.85 | 0.64 | 0.81 |
|  | Q3 | 0.86 | 0.75 | | 0.76 | 0.76 | 0.84 | 0.85 | 0.66 | 0.82 |
|  |  |  |  | |  |  |  |  |  |  |
| 4 | N | 955 | 955 | | 955 | 955 | 955 | 955 | 955 | 955 |
|  | MIN | 0.60 | 0.21 | | 0.21 | 0.21 | 0.42 | 0.48 | 0.24 | 0.52 |
|  | MEAN | 0.72 | 0.67 | | 0.68 | 0.70 | 0.88 | 0.62 | 0.63 | 0.64 |
|  | MAX | 0.79 | 1.88 | | 1.94 | 8.13 | 1.28 | 0.72 | 0.93 | 0.72 |
|  | STD | 0.03 | 0.23 | | 0.24 | 0.38 | 0.12 | 0.04 | 0.09 | 0.03 |
|  | MSD | 0.000 | 0.216 | | 0.228 | 0.888 | 0.042 | 0.012 | 0.015 | 0.007 |
|  | Q1 | 0.70 | 0.52 | | 0.53 | 0.53 | 0.80 | 0.60 | 0.57 | 0.62 |
|  | Median | 0.72 | 0.62 | | 0.63 | 0.63 | 0.89 | 0.62 | 0.64 | 0.64 |
|  | Q3 | 0.74 | 0.76 | | 0.77 | 0.77 | 0.96 | 0.65 | 0.70 | 0.66 |

^†^MSD=Mean squared deviation, Q1 is the lower quartile and Q3 is the upper quartile.* The number of the equation used in the text is in parenthesis.

**Table S2.** Descriptive statistics for the estimated true heritability assuming

**that genotypes are not correlated for each of the four scenarios.**

| Statistics | Scenario 1 | | Scenario 2 | |
| --- | --- | --- | --- | --- |
|  | Uncorrelated | Correlated | Uncorrelated | Correlated |
| MIN | 0.305 | 0.56 | 0.036 | 0.09 |
| MEAN | 0.476 | 0.82 | 0.148 | 0.73 |
| MAX | 0.627 | 0.71 | 0.313 | 0.42 |
| STD | 0.052 | 0.04 | 0.047 | 0.11 |
|  | Scenario 3 | | Scenario 4 | |
| MIN | 0.517 | 0.66 | 0.069 | 0.36 |
| MEAN | 0.587 | 0.79 | 0.129 | 0.63 |
| MAX | 0.644 | 0.73 | 0.195 | 0.52 |
| STD | 0.020 | 0.02 | 0.023 | 0.04 |


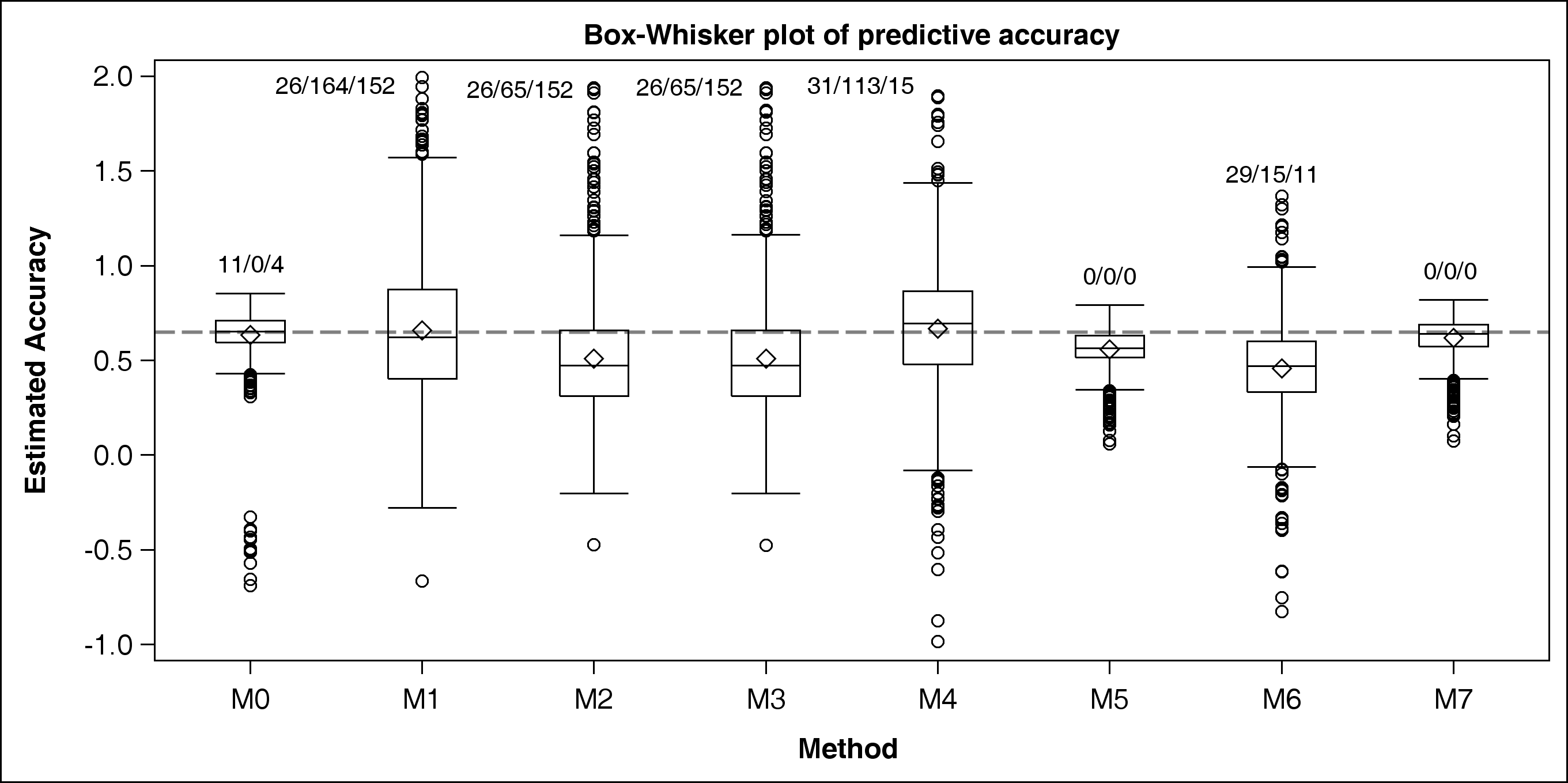


**Figure S1**. Box Whisker plot of all the predictive accuracies for scenario 2. The numeric labels (a/b/c) for each method denote the number of datasets out of the total of 1000 for which the estimated predictive accuracy was a) less than 0, b) greater than 1, c) the mixed model did not converge or heritability was equal to 0.

**
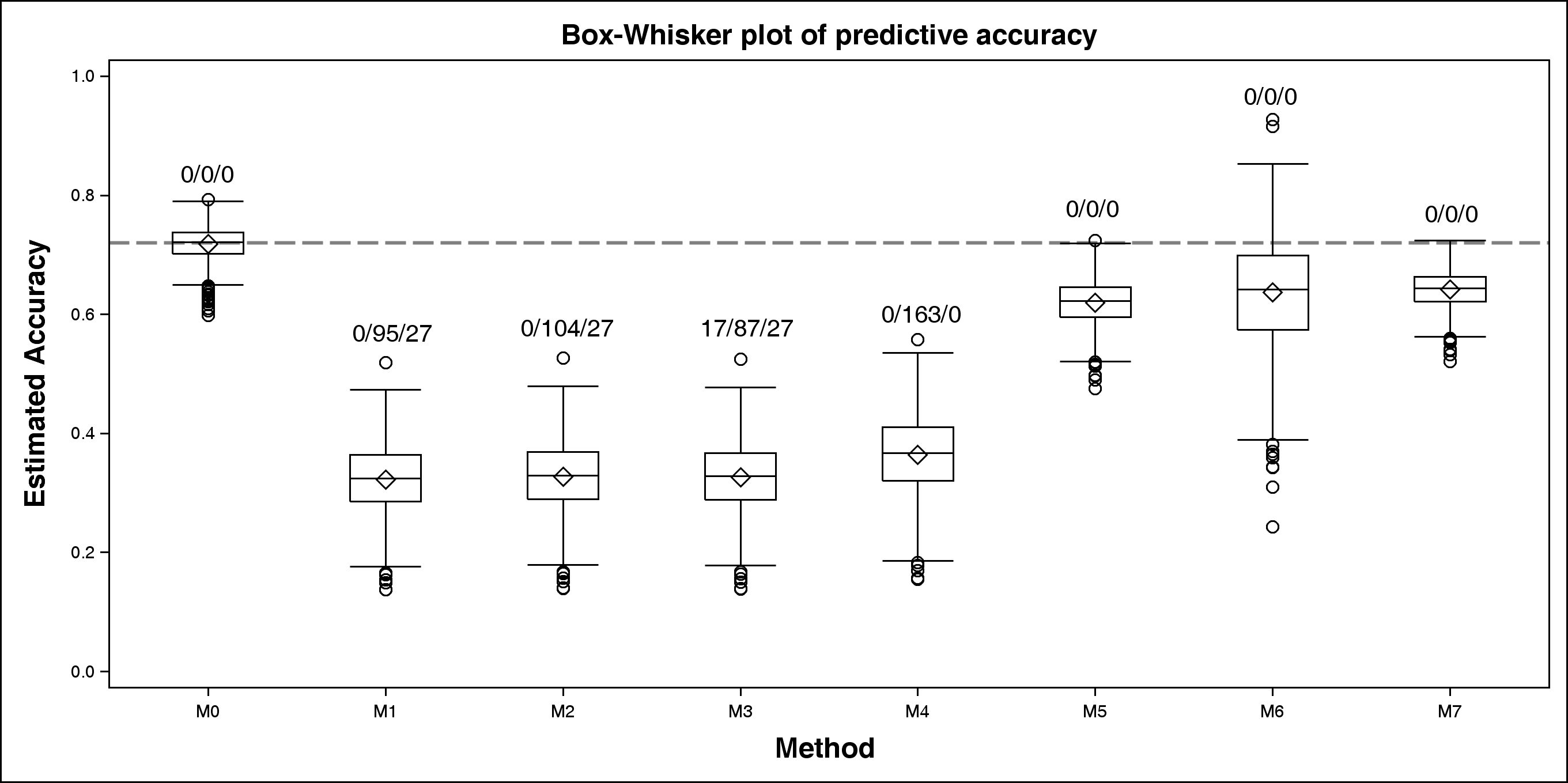
**

**Figure S2**. Box Whisker plot of all the predictive accuracies for scenario 4. The numeric labels (a/b/c) for each method denote the number of datasets out of the total of 1000 for which the estimated predictive accuracy was a) less than 0, b) greater than 1, c) the mixed model did not converge or heritability was equal to 0.

**
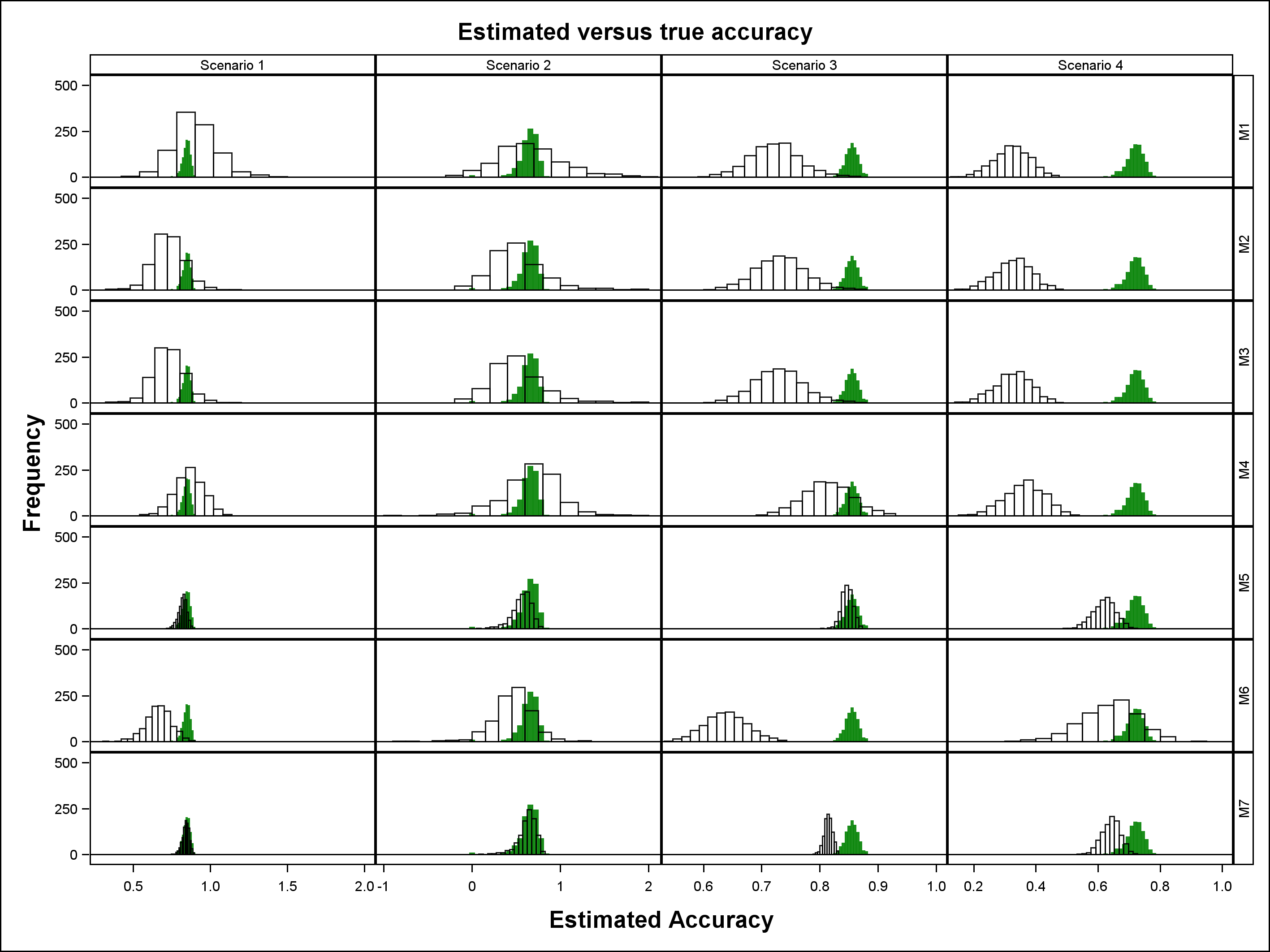
**

**Figure S3**. Frequency histograms for the true (green) versus the estimated (white) predictive accuracy for all the seven methods and four scenarios.


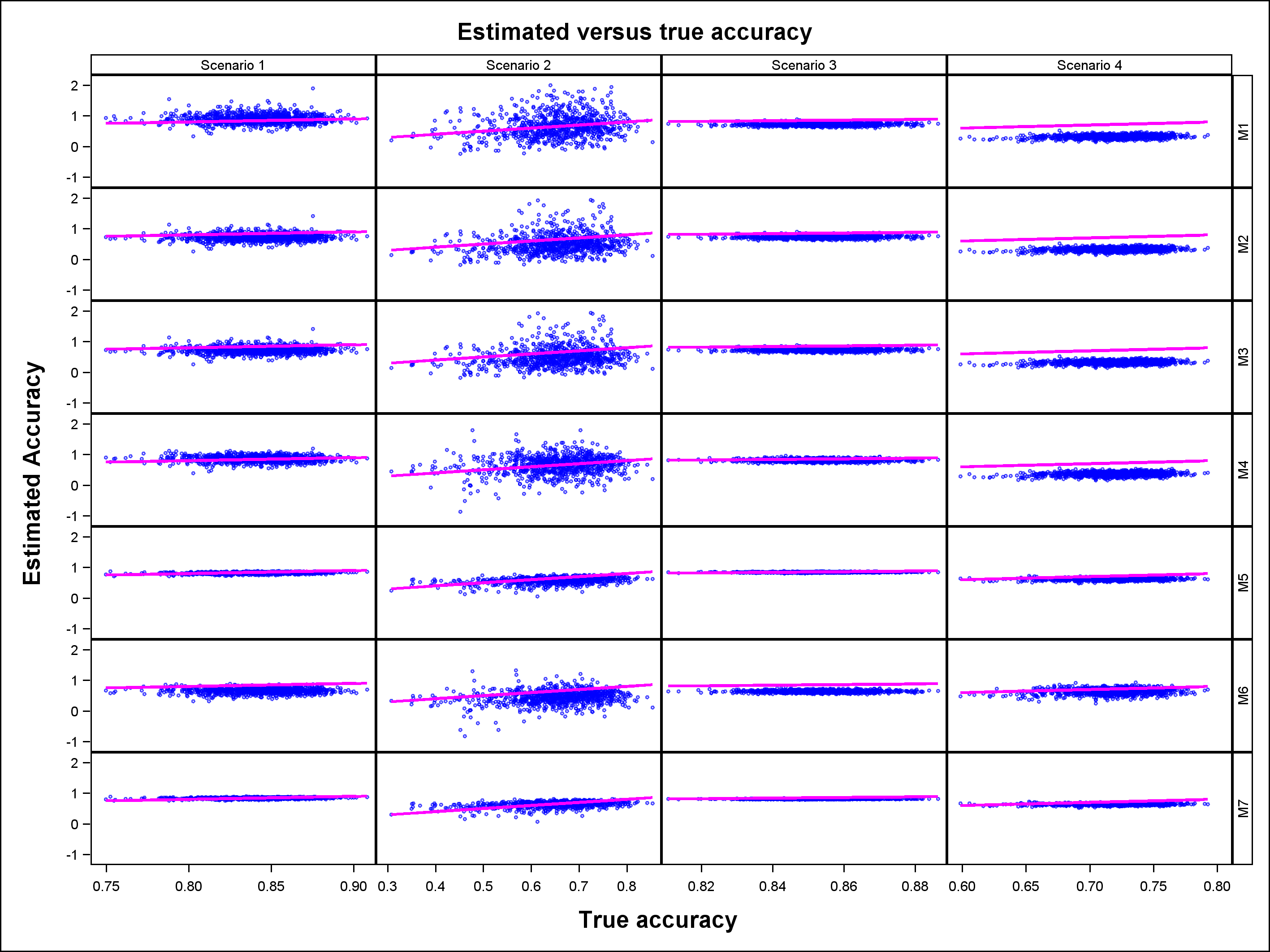


**Figure S4**. Scatter plots of estimated predictive accuracy against the true accuracy for all the seven methods and four scenarios. The 1:1 ($y=x$) line is superimposed for comparison.

**
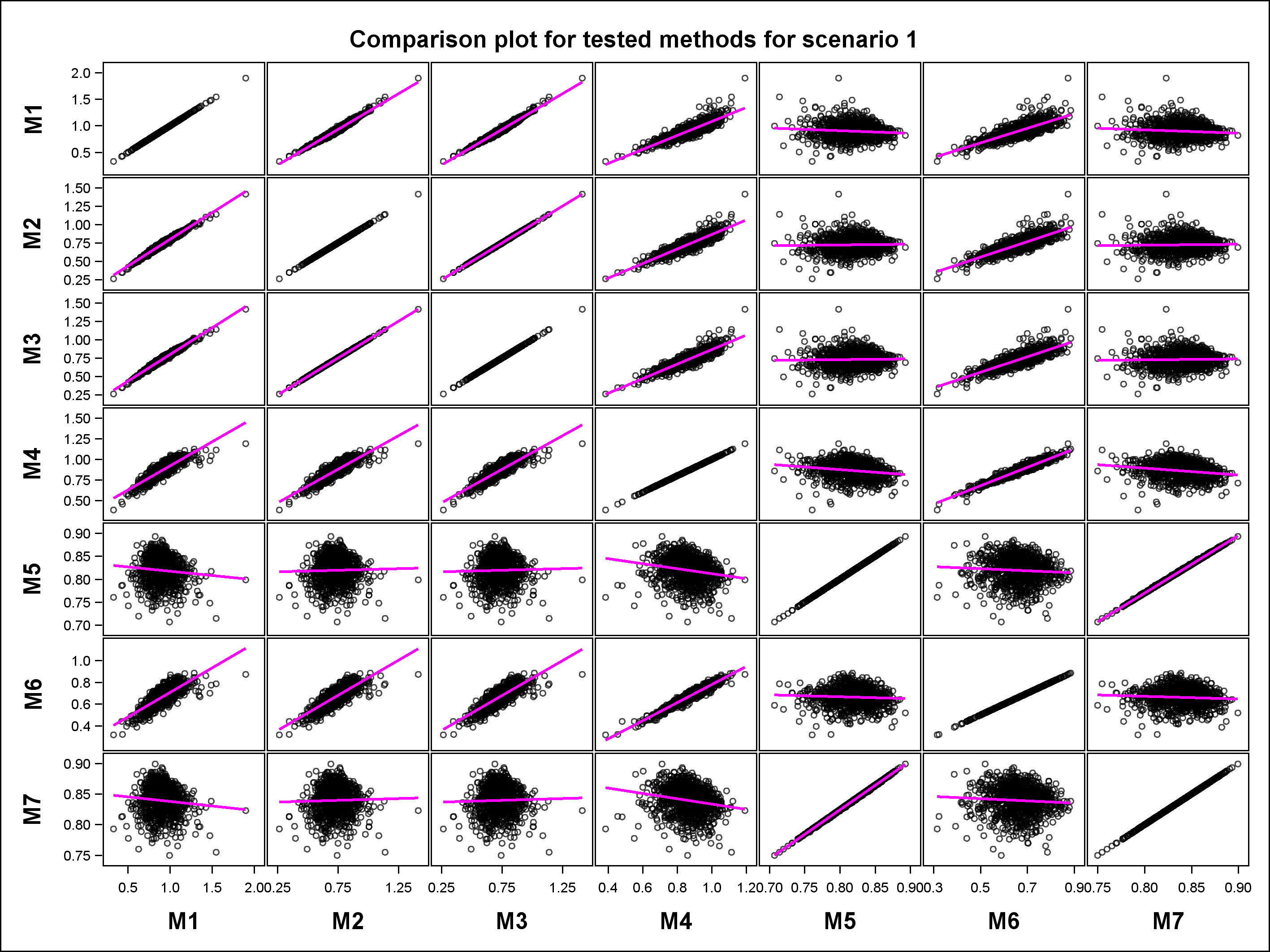
**

**Figure S5**. Scatter plots comparing the estimated predictive accuracies for pairs of the seven tested methods for scenario 1.

**
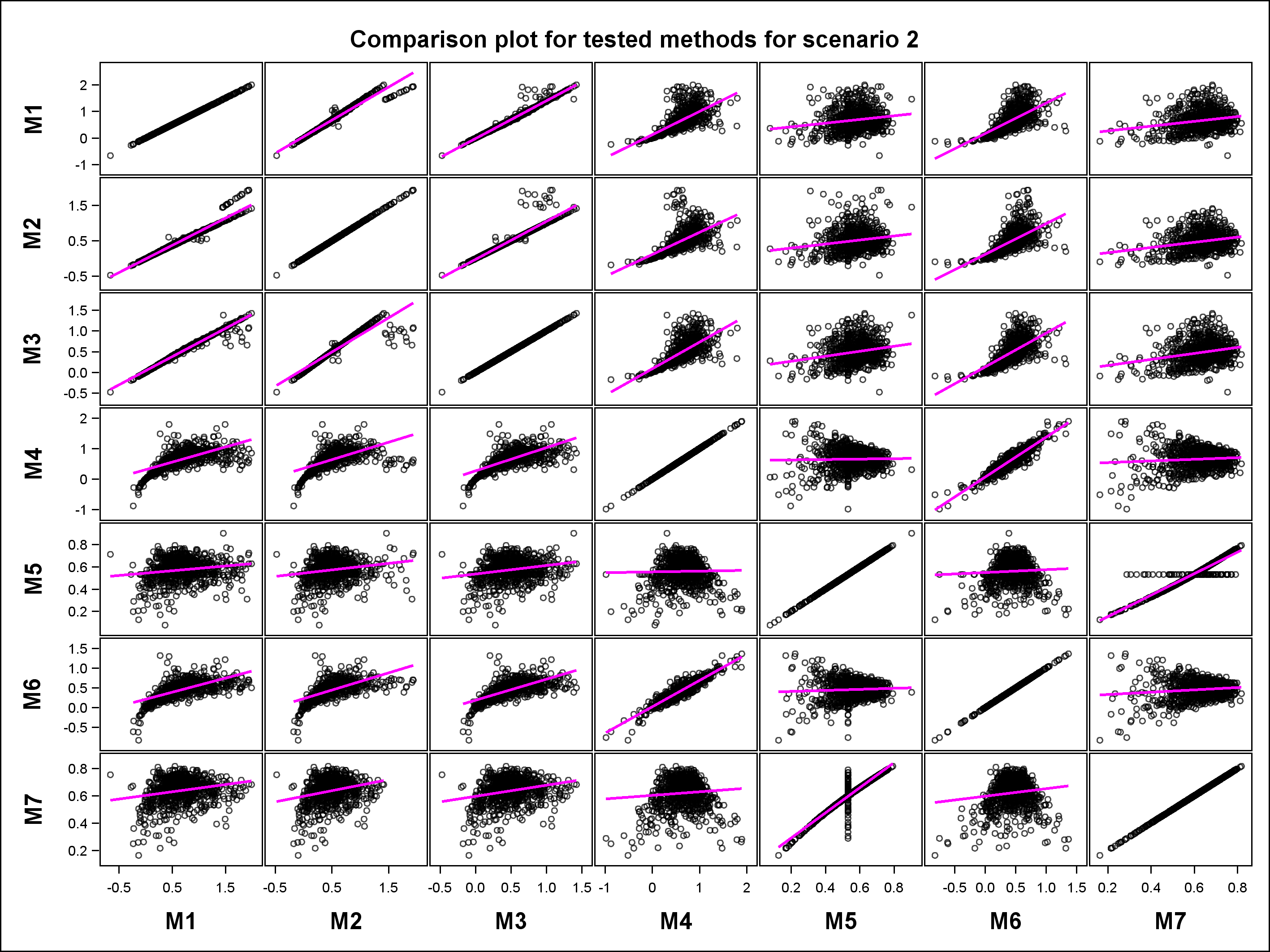
**

**Figure S6**: Scatter plots comparing the estimated predictive accuracies for pairs of the seven tested methods for scenario 2.

**
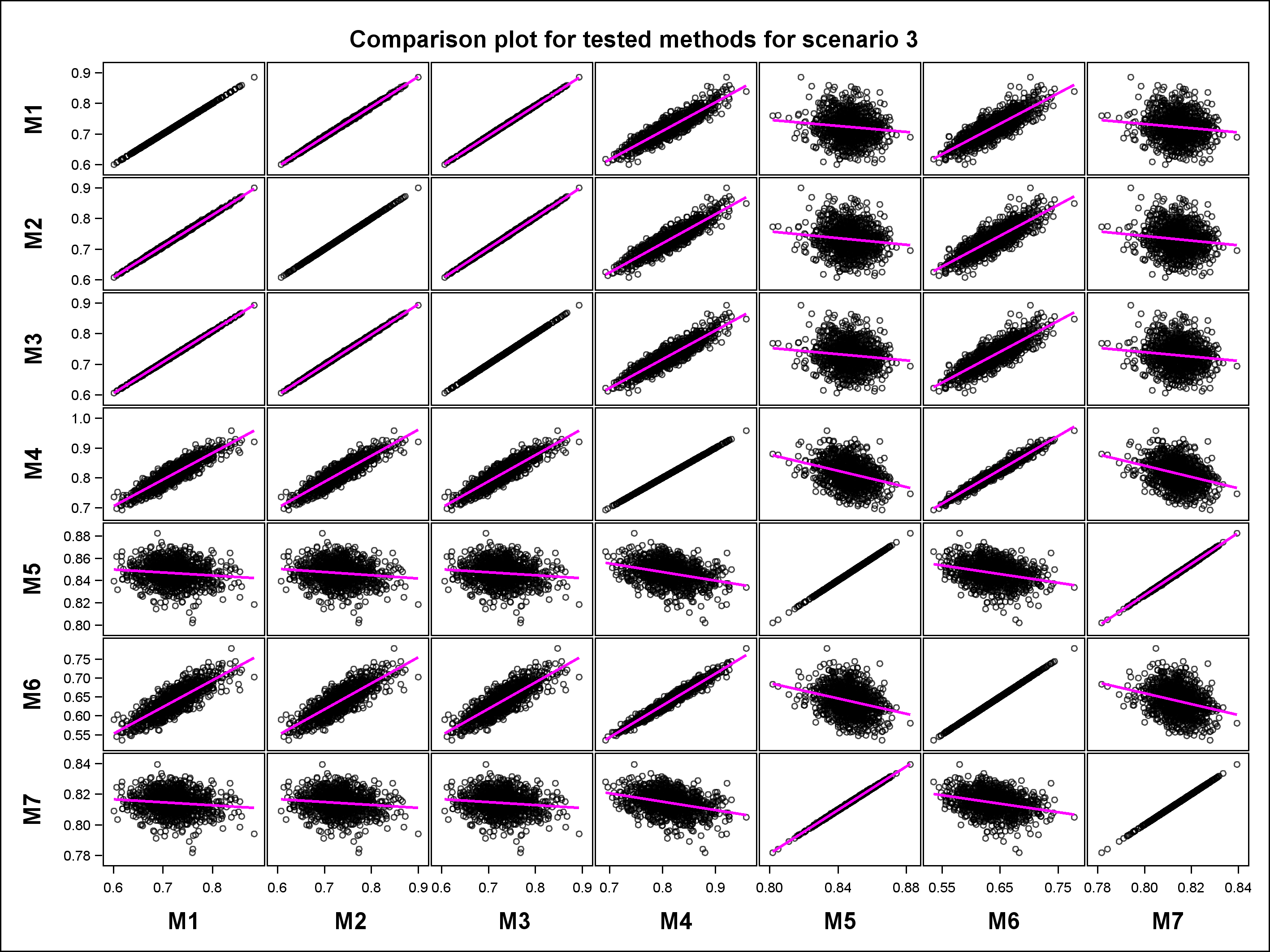
**

**Figure S7**: Scatter plots comparing the estimated predictive accuracies for pairs of the seven tested methods for scenario 3.


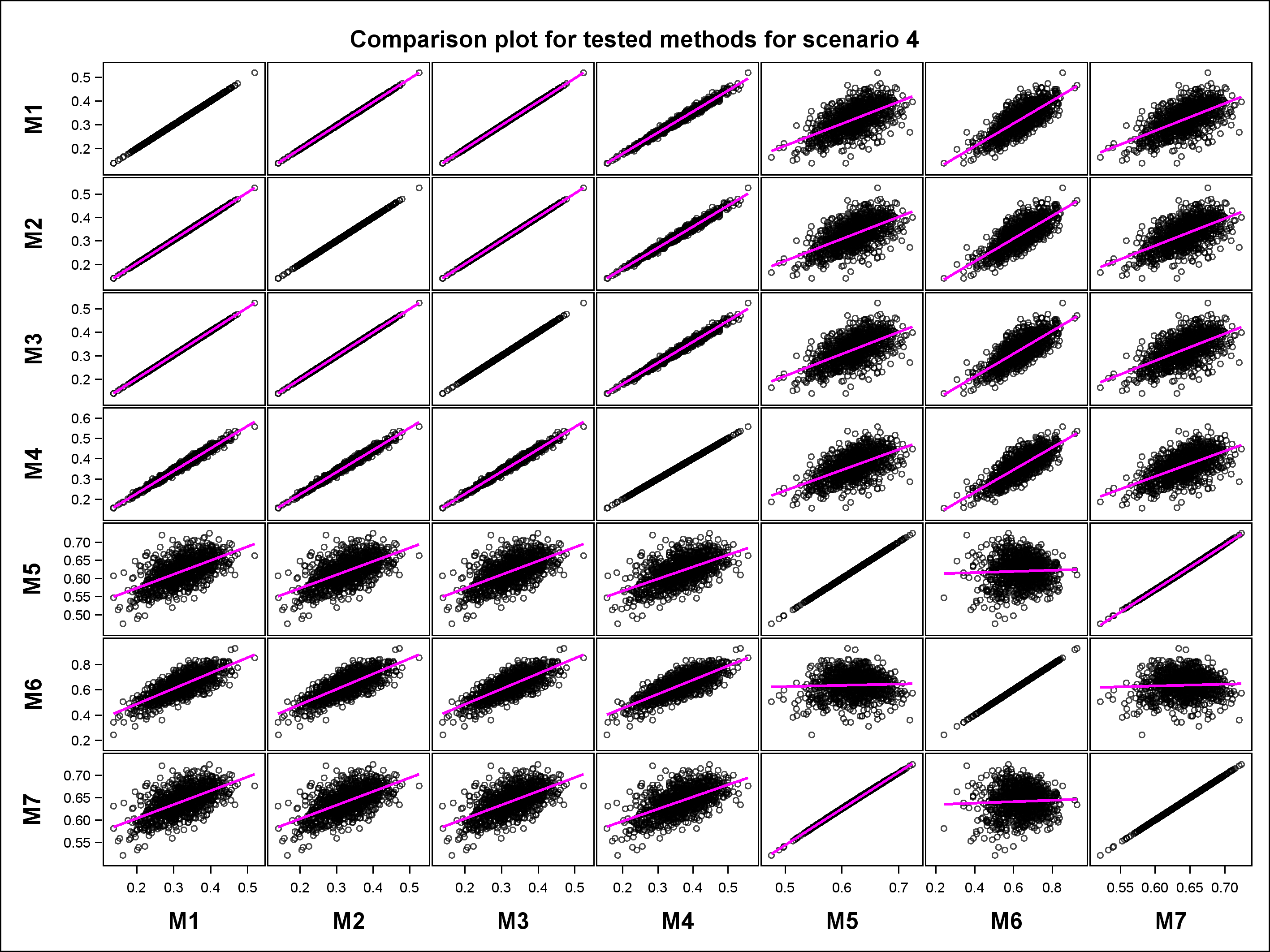


**Figure S8**: Scatter plots comparing the estimated predictive accuracies for pairs of the seven tested methods for scenario 4.
