# Supplementary material for: Evaluation of approaches for estimating the accuracy of genomic prediction in plant breeding
Source: BMC Genomics. 2013 Dec 6;14:860. doi: 10.1186/1471-2164-14-860 (PMC3879103; doi:10.1186/1471-2164-14-860)
Supplement: Additional file 4 — SAS (version 9.3) code used to simulate phenotypic data and implement all the seven methods. [file 1471-2164-14-860-S4.doc]

**SAS (Version 9.3) code used to simulate phenotypic data and implement all the seven methods**

/*----This code is based on the KWS dataset. This data set has 11646 markers, 9 trial, 10X10 blocks, 2 replicate and 698 genotypes. GRP, GENA, GENB, switch1 (appears in the text as $Z1$) and switch2 (appears in the text as $Z2$) are described in the main body of the paper.---*/

/*1. Calculate gamma=Z*t(Z) for use as ldata in proc mixed below---*/

**proc** **iml**;

use z;

read all var _NUM_ into z;/* reads a dataset called z containing markers */

gamma=z*t(z);

create gamma from gamma;

append from gamma;

**quit**;

/*Insert variables: row=_N_ and parm =1 in the gamma data file-----------*/

**data** gamma_ag; set gamma; row=_N_; parm=**1**; **run**;

**/*2.** Estimate marker (u), block (b) and error (e) variance components, assuming correlated

genotype effects. This is needed to simulate genetic effects as the sum of true breeding values, block and plot effects-*/

**proc** **mixed** data=maize_data maxiter=**1000** maxfunc=**5000** ;

ods output covparms=covparms_correlated_kws solutionR=solR;

class GRP GENA GENB loc REP BLOCK TRIAL ;

model GDYout=GRP ;

Random switch1*GENB/ldata=KWS_gamma type=lin(**1**);

Random TRIAL TRIAL*REP TRIAL*REP*BLOCK GRP*switch2*GENA/s ;

**run**;

/*3. Simulate random SNP effects as random draws from a normal distribution with zero mean and variance= marker variance (=var_u) estimated from the real marker dataset by proc mixed ----------*/

%let p=11646;

**data** u;

%let var_u=0.005892;

index=**1**;

array u u1-u&p;

do i=**1** to &p;

u[i]=normal(-**1**)*sqrt(&var_u);/*(-1) means use the current computer

time as the random number seed-*/

end;

output;

drop i;

**run**;

**proc** **sort** data=u;

by index;

**run**;

/*4. Generate genetic effects as true breeding values---*/

**data** z;

set z;

index=**1**;

**run**;

**proc** **sort** data= z;

by index;

**run**;

**data** z2;

set z;

geno_rnda=substr(labcode,**10**,**4**);

geno_rndb=input(geno_rnda,**8.**);

geno_rnd=_N_;

if geno_rnd<=**698**;

drop geno_rnda geno_rndb labcode;

**run**;

**proc** **sort** data= z2;

by index;

**run**;

**data** geno_eff ;

merge u z2 (sortedby=index);

by index;

array z z1-z&p;

array u u1-u&p;

g=**0**;

do i=**1** to **&p**;

g= g+z[i]*u[i];

end;

keep g geno_rnd;

**run**;

**proc** **sort** data=geno_eff ;

by geno_rnd;

**run**;

/*5. Simulate new data sets based on an alpha design using the block and error variance

components estimated by Proc Mixed above. The simulated datasets are assumed to come from

a normal distribution, with mean=0 and variance=block variance------------------------------------*/

**data** alphad4; set alphad3;

where geno_rnd ne **.**;

**run**;

%let sim_max=1000;

**%macro** sim_data(sim);

%let var_b=6.3148;

%let var_e=53.8715;

%do k=**1** %to &sim_max %by **1**;

data alpha;

sd_block=sqrt(&var_b); *Standard deviation of block variance;

sd_plot =sqrt(&var_e); *Standard deviation of plot variance;

do sim =**1** to &sim_max; *Number of replicates=Number of simulated data sets;

do i=**1** to n; *Sample size=number of genotypes;

set alphad4 nobs=n point=i;

if plot=**1** then block_eff=sd_block*normal(**1000**+%eval(&sim));

plot_eff=block_eff+sd_plot*normal(**1000**+%eval(&sim));

output;

end;

end;

stop;

run;

%end;

**%Mend** ;

%***sim_data***(sim=&sim_max);

**proc** **sort** data=alpha;

by geno_rnd;

**run**;

/*Merge the simulated block and plots effects with genetic effects to obtain a complete

data set with simulated phenotypic effects for use in the models below-------------------------*/

**data** geno_eff2;

set geno_eff;

geno_rnd2=_N_;

drop geno_rnd;

**run**;

**proc** **sort** data=geno_eff2;

by geno_rnd2;

**run**;

**data** alpha_1;

set alpha(rename=(geno_rnd=geno_rnd2));

**run**;

**proc** **sort** data=alpha_1;

by geno_rnd2;

**run**;

**data** alpha2;

merge alpha_1 geno_eff2;

by geno_rnd2;

yield=plot_eff+g;

**run**;

**proc** **sort** data=alpha2(rename=(geno_rnd2=geno_rnd));

by sim geno_rnd rep;

**run**;

/* Replicate gamma matrix sim_max (=1000) times, for use in by processing below*/

**data** gamma_ag2;

set gamma_ag;

row=_N_; parm=**1**; /*---Insert row number and parm ID -----*/

if row<=**698**;

**run**;

**data** gamma_all;

set gamma_ag2;

do sim=**1** to &sim_max;

output;

end;

**run**;

**proc** **sort** data=gamma_all;

by sim;

**run**;

/* End Replicate matrix-------------------------------------------*/

/*6. Compute Lsmeans for genotypes, which are later used as phenotypic data in cross validation. The fitted model uses PV=yield as a response and estimates the variance-covariance matrix for the lsmeans, which is used to compute heritability in Method 2 (*ad hoc* measure based on BLUE)-------------------------*/

ods output lsmeans=lsmean_m2 diffs=lsmeansdiffs_m2;

**proc** **hpmixed** data=alpha2;

by sim;

class rep block geno_rnd;

model yield=geno_rnd;

random rep rep*block;

lsmeans geno_rnd/pdiff cov;

**run**;

**data** lsmeans_m2;

set lsmean_m2;

keep estimate geno_rnd sim;

**run**;

**proc** **sort** data=lsmeans_m2;

by sim geno_rnd;

**run**;

/*7. Compute Methods 1, 2 and 3 assuming genotypes are uncorrelated---*/

/*Compute the genetic variance using Methods 1, 2 and 3 and mixed

model equations used to compute Method 3 using the *ad hoc* measure based on BLUP-*/

ods output covparms=cp_raw_m1m2m3 g=g_raw mmeqsol=mmeqsol_raw_m3;

**proc** **mixed** data=alpha2 lognote mmeqsol mmeq maxiter=**1000**;

by sim;

class rep block geno_rnd;

model yield=;

random geno_rnd/g;

random rep*block;

**run**;

/* Method 1 starts: Compute the standard heritability --*/

**data** var_g_m1m2m3 (rename=(estimate=var_g));

set cp_raw_m1m2m3;

where CovParm="geno_rnd";

**run**;

**data** var_e_m1 (rename=(estimate=var_e));

set cp_raw_m1m2m3;

where CovParm="Residual";

**run**;

**data** H2_stand_m1;

merge var_g_m1m2m3 (sortedby=sim) var_e_m1 (sortedby=sim);

by sim;

H2_stand=var_g/(var_g+**0.5***var_e);

keep sim H2_stand;

**run**;

/* End of computation of the standard heritability (Method 1)--*/

/* Method 2 starts : Compute heritability using *ad hoc* measure based on BLUE----*/

**data** diffs;

set lsmeansdiffs_m2;

half_var_lsmeans=**0.5***stderr****2**;

**run**;

**proc** **means** data=diffs mean;

by sim;

var half_var_lsmeans;

output out=half mean=;

**run**;

**data** H2_blue_m2;

merge var_g_m1m2m3(sortedby=sim) half(sortedby=sim);

by sim;

H2_blue_m2=var_g/(var_g+half_var_lsmeans);

keep sim H2_blue_m2;

**run**;

/*End of Computation of heritability using the *ad hoc* measure based on BLUE (Method 2)*/

/* Method 3 starts: Estimate heritability using *ad hoc* measure based on BLUP---*/

/* Compute the average variance of a difference from the variance-covariance matrix of adjusted means*/

/* This macro breaks up the 1000 simulated datasets into blocks of size 100 hundred to speed

up reading the data sets*/

**%macro** adhoc_blup(sim);

%do s=**0** %to **9** %by **1**;

%do k=**1** %to **100** %by **1**;

%let p=%eval((&s+1)*100);

%let l=%eval((&k+(&s*100)));

data mmeqsol_raw_&p ;

set mmeqsol_raw_m3;

where %eval(&s***100**)<sim<=&p;

run;

proc iml;

one=j(**698**,**1**,**1**);

create one from one;

append from one;

use mmeqsol_raw_&p ;

read all var _NUM_ where(sim=&l) into C22 ;

c22_1=C22[**4**:**701**, **9**:**706**];

create C22_1 from C22_1;

append from C22_1;

sum_&l=t(one)*C22_1[, **1**:ncol(C22_1)]*one;

create work.sum_&l from sum_&l;

append from sum_&l;

s=**2**/(**698*****697**);

create s from s;

append from s;

trace_&l=trace(C22_1[, **1**:ncol(C22_1)]);

create work.trace_&l from trace_&l;

append from trace_&l;

var_mean_&l=s*(**698***trace_&l-sum_&l);

create work.var_mean_&l from var_mean_&l;

append from var_mean_&l;

use var_g_m1m2m3;

read all var _NUM_ where(sim=&l) into var_g2 ;

if var_g2[**2**] > **0** then H2_blup=**1**-var_mean_&l/(**2***var_g2[**2**]);

else if var_g2[**2**]<=**0** then H2_blup=**.**;

create H2_blup from H2_blup;

append from H2_blup;

data H2_blup2;

set H2_blup (rename=(col1=H2_m3));

sim=&l;

run;

quit;

proc append data =H2_blup2 base=H2_blup_m3;

run;

%end;

%end;

**%Mend** adhoc_blup;

%***adhoc_blup***;

**proc** **sort** data=H2_blup_m3 out=H2_blup_m3_final nodupkey;

by sim;

**run**;

/*End of Estimation of heritability using the *ad hoc* measure based on BLUP (Method 3)*/

/*8. Estimate breeding values (g_hat) using the simulated data and use these breeding values to compute

the correlation between (1) the predicted (g_hat) and the true (g) breeding values (as a benchmark

=true correlation) and (2) the correlation between the predicted and the simulated phenotypic values-*/

/*Use only simulated phenotypic data, assuming correlated genotypes*/

/*RRBLUP for Methods 4, 5 and 7: assuming genotypes are correlated----*/

/*and used to compute true correlation---*/

ods output covparms=cp_m4m5m7 G=G_m4m5m6 mmeqsol=mmeqsol_m7 solutionr=ghat_m0;

**proc** **mixed** data=alpha2 lognote mmeqsol mmeq;

by sim;

class rep block geno_rnd;

model yield=rep;

random geno_rnd/g solution sub=int type=lin(**1**) ldata=gamma_all;

random rep*block/ solution;

**run**;

/* Used for Methods 4, 5 and 6*/

**data** G_m4m5m6;

set G_m4m5m6;

where effect="geno_rnd";

keep sim col1-col698;

**run**;

/* Used for methods 4, 5 and 7------------------------------------------ */

/* Compute variances of TBV (g) needed to estimate standard heritability*/

**data** var_g_m7;

set cp_m4m5m7 (where=(CovParm='LIN(1)') rename=(estimate=var_g));

keep var_g sim;

**run**;

/* Used for methods 4, 5 and 7---------------------------------------*/

**data** var_e_m4m5;

set cp_m4m5m7 (where=(CovParm='Residual') rename=(estimate=var_e));

keep var_e sim;

**run**;

/* Merge estimated genetic effects (g_hat) and true genetic effects (g)

to compute the true correlation (Method 0)*/

**proc** **sort** data=ghat_m0;

by geno_rnd ;

where effect='geno_rnd';

**run**;

**data** g_ghat;

merge geno_eff2(rename=(geno_rnd2=geno_rnd)) ghat_m0 (rename=(estimate=ghat));

by geno_rnd;

**run**;

**proc** **sort** data=g_ghat;

by sim ;

**run**;

/* True correlation: Compute the correlation between GBV and TBV

(corr_GBV_TBV=cov(GBV,TBV)/sqrt(var_GBV*var_TBV)) using the simulated datasets*/

ods output pearsoncorr=corr_GBV_TBV_sim (drop=Pg_hat);

**proc** **corr** data=g_ghat cov nosimple pearson;

by sim;

var ghat ;

with g;

**run**;

/* Compute true heritability from the true correlation between the predicted

and the true breeding values*/

**data** H2_real;

set corr_GBV_TBV;

H2_real=(ghat)****2**;

keep sim H2_real;

**run**;

/*9. New method (4) used to estimate heritability-----------------*/

/* This macro breaks up the 1000 simulated datasets in to blocks

of seize 100 hundred to speed up reading the data set*/

**%macro** ***H2_m4***;

%do s=**0** %to **9** %by **1**;

%do k=**1** %to **100** %by **1**;

%let p=%eval((&s+1)*100);

%let l=%eval((&k+(&s*100)));

data G_&p;

set G_m4m5m6;

where %eval(&s***100**)<sim<=&p;

run;

proc iml;

/* Compute Method 4 starts/

/*Compute P_u --------------*/

one_r=i(**698**);

create one_r from one_r;

append from one_r;

i=i(**698**);

create i from i;

append from i;

j=j(**698**,**698**,**1**);

create j from j;

append from j;

P_u=(i-((**1**/**698**)*j))/(**698**-**1**);

create P_u from P_u;

append from P_u;

/*---End: P_u----------------------------*/

/* Compute error variance (var_e)*/

use var_e_m4m5;

read all var _NUM_ into cp_r where (sim=&l);

/*-------End var_e------------------*/

/*Compute the variance-covariance of error R*/

R_&l=cp_r[**2**]*one_r;

create R_&l from R_&l;

append from R_&l;

traceR=trace(R_&l*P_u);

create traceR from traceR;

append from traceR;

/* End of R----------------------------------*/

/* Compute the variance-covariance of G */

use G_&p;

read all var _NUM_ where(sim=&l) into g3;

create g3 from g3;

append from g3;

/* ------End G--------------------------*/

/*-Compute the trace of G --- --------*/

H_trace=trace(P_u*g3[, **2**:ncol(g3)]);

create H_trace from H_trace;

append from H_trace;

data H_trace_2;

set H_trace;

sim=&l;

run;

data traceR2;

set traceR;

sim=&l;

run;

proc append data=traceR2 base=traceR_final;

run;

proc append data=H_trace_2 base=H_trace_final;

run;

quit;

%end;

%end;

**%mend**;

%***H2_m4***;

**data** H_trace_final2;

set H_trace_final (rename=(col1=traceG));

**run**;

**data** traceR_final2;

set traceR_final (rename=(col1=traceR));

**run**;

**data** H2_m4;

merge H_trace_final2(sortedby=sim) traceR_final2(sortedby=sim);

H2_m4=traceG/(traceG + traceR);

keep sim H2_m4;

**run**;

/*-- Method 4 ends------------------------------------------------------*/

/*10. New method (5) used to estimate heritability and predictive accuracy*/

/* This macro breaks up the 1000 simulated datasets in to blocks

of seize 100 hundred to speed up reading the data set*/

**%macro** ***H_m5***;

%do s=**0** %to **9** %by **1**;

%do k=**1** %to **100** %by **1**;

%let p=%eval((&s+1)*100);

%let l=%eval((&k+(&s*100)));

data G_&p;

set G_m4m5m6;

where %eval(&s***100**)<sim<=&p;

run;

proc iml;

/* Method 5 starts: Compute H_m5 as a direct estimate of predictive accuracy*/

/* Compute variance-covariance G used ------*/

use G_&p;

read all var _NUM_ where(sim=&l) into g3;

create g3 from g3;

append from g3;

/* ------End G------------------------------------*/

/*Compute P2 ----------------*/

P2=(i-((**1**/**698**)*j));

create P2 from P2;

append from P2;

/*---End: P2---------------*/

/* Compute V, Q and C */

V_&l= g3[, **2**:ncol(g3)] + R_&l;

create V_&l from V_&l;

append from V_&l;

one_q=j(**698**, **1**, **1**);

create one_q from one_q;

append from one_q;

Q_&l=i-one_q*inv(t(one_q)*inv(V_&l)*one_q)*t(one_q)*inv(V_&l);

create Q_&l from Q_&l;

append from Q_&l;

C_&l=g3[, **2**:ncol(g3)]*inv(V_&l)*Q_&l;

create C_&l from C_&l;

append from C_&l;

H_m5=trace(P2*C_&l*g3[, **2**:ncol(g3)])/sqrt(trace(P2*g3[, **2**:ncol(g3)])

*trace(t(C_&l)*P2*C_&l*V_&l));

create H_m5 from H_m5;

append from H_m5;

data H_m5_2;

set H_m5 (rename=(col1=H_m5));

sim=&l;

run;

proc append data =H_m5_2 base=H_m5_final;

run;

quit;

%end;

%end;

**%mend**;

%***H_m5***;

/* Compute predictive accuracy using Method 5 ends */

/* Compute heritability using Method 5 starts */

**data** H2_m5;

set H_m5_final;

H2_m5=(H_m5)****2**;

keep sim H2_m5;

**run**;

/* Computation of heritability using Method 5 ends */

**/*11.** *k*-fold cross-validation (CV), where *k*=3------------------------------------------------------*/

/*---Macro is needed to create a table, in which the dataset is replicated k times

This is then merged with the actual dala-----------------------------------------------*/

/*-------Simum is the total number of different simulated datasets-------------*/

/*--------n_fold is the number of folds=3---------------------------------*/;

/*--------repnum is the number of replications of cross-validation=5------------*/;

/*--------Number_genotype is serial number of genotypes in each complete dataset=698----*/

%global foldnum repnum simnum;

**%macro** cvalid (Genotypenum, foldnum, repnum, simnum);

%do sim=**1** %to &simnum;

%do rep=**1** %to &repnum;

*----Use proc plan to do select random subsamples for each replicate---*;

proc plan seed=%eval(**600**+**100***&rep+**200***&sim);/**---Set the seed for random number generator---*/;

factors Subject =&Genotypenum

Order = **1** ordered;

treatments subsample = &foldnum perm;

output out=subsample1;

run;

*---Insert relicate, sim, type and fold in each fold of each replicate and simulated data-----*;

**%macro** ***fold***;

%do i=**1** %to &foldnum;

data subsample2_&i; set subsample1(in=j);

if j then replicate=&rep;

if j then sim=&sim;

if subsample eq &i then do; type='validation'; fold=&i; output; end;

else if subsample ne &i then do; type='training'; fold=&i; output; end;

run;

*--------------------------------------------------;

* Append each new dataset subsample2 to subsample3 ;

*--------------------------------------------------;

Proc append data=subsample2_&i base= subsample3; run;

%end;

**%mend**;

%***fold***;

%end;

%end;

proc datasets; delete subsample2_:; run; quit;

proc sort data= subsample3(rename=(subject=geno_rnd)); by sim replicate fold subsample type; run;

**%mend**;

%***cvalid*** (Genotypenum=**698**, foldnum=**3**, repnum=**5**, simnum=**1000**);

**data** lsmeans;

set lsmean_m2;

keep sim geno_rnd estimate;

**run**;

**proc** **sort** data= lsmeans;/*-------File with observed data------*/;

by sim geno_rnd;

**run**;

**proc** **sort** data= subsample3;

by sim geno_rnd;

**run**;

**data** subsample3;

merge subsample3 lsmeans;

by sim geno_rnd;

if type="validation" then yield_validation=estimate;

else if type="training" then yield_training=estimate;

**run**;

**proc** **sort** data= subsample3 out= subsample3;

by sim replicate fold subsample geno_rnd;

**run**;

/------------------------------End of cross validation--------------------------------------------------------/

/* **12**. Second stage of two-stage analysis using cross-validation--------------------------------*/

/*RR-BLUP for each replicate: will save estimate of yield (pred_cv) and

predicted breeding values (ghat) needed to compute method 6 and predictive ability:

This is used by methods 1, 2, 3 and 4 to compute predictive accuracy ---------------------*/

/*Replicate matrix and sim_max (1000) times,

replicate (=5) and fold (=3), for use in by processing*/

**data** all_gamma_cv;

set gamma_ag2;

do sim= **1** to **1000**;

do replicate=**1** to **5**;

do fold=**1** to **3**;

output;

end;

end;

end;

**run**;

/* This macro breaks up the 1000 simulated datasets into blocks

of size 50 to speed up reading the data sets*/

**%macro** ***cv***;

%do s=**0** %to **19** %by **1**;

%let p=%eval((&s+1)*50);

data subsample_&p;

set subsample3;

where %eval(&s***50**)<sim<=&p;

run;

proc sort data=subsample_&p;

by sim replicate fold;

run;

data gamma_&p;

set all_gamma_cv;

where %eval(&s***50**)<sim<=&p;

run;

proc sort data=gamma_&p;

by sim replicate fold;

run;

ods output solutionr=ghat_cv_&p convergencestatus=converge_cv_&p;

proc mixed data=subsample_&p maxiter=**1000** lognote maxfunc=**5000**;

by sim replicate fold;

class geno_rnd;

model yield_training=/outp=pred_cv_&p ;

random geno_rnd/sub=int type=lin(**1**) ldata=gamma_&p solution;

run;

%end;

**%mend**;

%***cv***;

**data** pred_cv;

merge pred_cv_&p ;

by sim;

**run**;

**data** ghat_cv;

merge ghat_cv_&p;

by sim;

**run**;

/*13. Compute predictive ability: needed for Methods 1, 2, 3 and 4

to calculate predictive accuracy--------------------------------------*/

**proc** **sort** data=pred_cv;

by sim replicate fold geno_rnd;

**run**;

**proc** **sort** data=ghat_cv ;

by sim replicate fold geno_rnd;

**run**;

**data** ghat_cv_yield_valid ;

merge ghat_cv (rename=(estimate=ghat))

pred_cv(rename=(estimate=yield_hat));

by sim replicate fold geno_rnd;

**run**;

**proc** **sort** data=ghat_cv_yield_valid;

by sim replicate fold;

**run**;

ods output cov=cov_gbv_pv_cv pearsoncorr=corr_gbv_pv_cv;

**proc** **corr** data=ghat_cv_yield_valid cov nosimple pearson;

by sim replicate fold;

where type='validation';

var ghat;

with yield_validation;

**run**;

**proc** **means** data=cov_gbv_pv_cv nway noprint;

class sim;

var ghat;

output out=mean_cov_gbv_pv_cv(drop=_TYPE_ _FREQ_) mean=;

**run**;

**proc** **means** data=corr_gbv_pv_cv nway noprint;

class sim;

var ghat;

output out=mean_corr_gbv_pv_cv(drop=_TYPE_ _FREQ_) mean=;

**run**;

/* End of computation of predictive ability --------------------------------*/

/*14. Compute of Method 6 starts ------------------------------------------*/

/* Variances of GBV, yield and TBV----------------------------------------*/

**proc** **means** data=ghat_yield_valid_cv nway noprint;

class sim replicate fold;

where type="validation" and status=**0**;

var ghat Yield_validation;

output out=variances_cv(drop=_type_ _freq_) Var=;

**run**;

**proc** **means** data=variances_cv nway noprint;

class sim;

var ghat yield_validation;

output out=mean_variances_cv(drop=_TYPE_ _FREQ_) mean=;

**run**;

**data** variances_gbv;

set mean_variances_cv;

keep sim ghat;

**run**;

**data** cov_gbv ;

set mean_cov_gbv_pv_cv(rename=(ghat=cov));

keep sim cov;

**run**;

/*Directly estimate the correlation between the predicted and the true breeding values using method 6 */

**%macro** ***EW_m6***;

%do s=**0** %to **9** %by **1**;

%do k=**1** %to **100** %by **1**;

%let p=%eval((&s+1)*100);

%let l=%eval((&k+(&s*100)));

data G_&p;

set G_m4m5m6;

where %eval(&s***100**)<sim<=&p;

run;

proc iml;

i=i(**698**);

create i from i;

append from i;

j=j(**698**,**698**,**1**);

create j from j;

append from j;

M=i-(**1**/**698**)*j;

create M from M;

append from M;

use Cov_GBV ;

read all into cov_cv;

create cov_cv from cov_cv;

append from cov_cv;

use variances_GBV ;

read all var _NUM_ into var_GBV;

create var_GBV from var_GBV;

append from var_GBV;

use G_&p;

read all var _NUM_ where(sim=&l) into g3;

create g3 from g3;

append from g3;

MG_&l=M*g3[, **2**:ncol(g3)];

create MG_&l from MG_&l;

append from MG_&l;

E_var_TBV=(**1**/(**698**-**1**))*trace(MG_&l);

create E_var_TBV from E_var_TBV;

append from E_var_TBV;

data work.E_var_TBV2;

set work.E_var_TBV;

sim=&l;

run;

quit;

proc append data =E_var_TBV2 base=E_var_TBV_final;

run;

%end;

%end;

**%Mend**;

%***EW_m6***;

**proc** **sort** data=E_var_TBV_final;

by sim;

**run**;

**data** m6_s4;

merge E_var_TBV_final(rename=(col1=E_var_tbv)) variances_GBV Cov_GBV;

by sim;

keep sim E_var_tbv cov ghat;

**run**;

**data** corr_gbv_tbv_m6;

set m6_s4;

if (ghat*E_var_tbv>**0**) then corr_gbv_tbv_m6=cov/(sqrt(ghat*E_var_tbv));

keep sim corr_gbv_tbv_m6;

**run**;

/*End of computations for Method6------------------------------------------------------*/

/*15. Directly estimate the correlation between the predicted and the true breeding value using Method 7. This macro breaks up the 1000 simulated datasets into blocks of size 100 hundred to speed up reading the data sets */

**%macro** ***m7***;

%do s=**0** %to **9** %by **1**;

%do k=**1** %to **100** %by **1**;

%let p=%eval((&s+1)*100);

%let l=%eval((&k+(&s*100)));

data gamma_&p;

set gamma_all;

where %eval(&s***100**)<sim<=&p;

run;

data mmeqsol_&p;

set mmeqsol_m7;

where %eval(&s***100**)<sim<=&p;

run;

proc iml;

use var_g_m7;

read all var _NUM_ where(sim=&l) into var_g22;

use gamma_&p;

read all var _NUM_ where(sim=&l) into gamma3;

Gu=vecdiag(gamma3)*var_g22[**2**];

create Gu from Gu;

append from Gu;

use mmeqsol_&p;

read all var _NUM_ where(sim=&l) into C22 ;

c22_1=C22[**4**:**701**, **9**:**706**];

create C22_1 from C22_1;

append from C22_1;

M= Gu-C22_1;

create M from M;

append from M;

r_hpp=sqrt(vecdiag(M)/Gu);

create r_hpp from r_hpp;

append from r_hpp;

r_mean=mean(r_hpp);

create r_mean from r_mean;

append from r_mean;

data r_mean2;

set r_mean;

sim=&l;

run;

quit;

proc append data =r_mean2 base=corr_M7;

run;

%end;

%end;

**%mend**;

%***m7***;

/*------------End of computations for Method 7------------------------------------------------------------*/

/* 16. Computed predictive accuracy for all the seven methods-------------------------------------*/

**data** predictive_accuracy;

merge corr_GBV_tbv_sim mean_corr_gbv_pv_cv (rename=(ghat=pred_ab)) H2_stand_m1 H2_blue_m2

H2_blup_m3_final H2_m4 H_m5_final corr_gbv_tbv_m6 corr_m7 (rename=(col1=corr_m7));

by sim;

pred_ac_stand_m1 = pred_ab /sqrt(H2_stand);

pred_ac_blue_m2 = pred_ab /sqrt(H2_blue_m2);

pred_ac_blup_m3 = pred_ab /sqrt(H2_m3);

pred_ac_m4 = pred_ab/sqrt(H2_m4);

keep sim corr_real pred_ac_stand_m1 pred_ac_blue_m2 pred_ac_blup_m3

pred_ac_m4 H_m5 corr_gbv_tbv_m6 corr_m7;

**run**;

/* End of the SAS code-------------------------------------------------------------*/
